# OCT4 supports extended LIF-independent self-renewal and maintenance of transcriptional and epigenetic networks in embryonic stem cells

**DOI:** 10.1038/s41598-017-16611-y

**Published:** 2017-11-27

**Authors:** Runsheng He, Besa Xhabija, Batool Al-Qanber, Benjamin L. Kidder

**Affiliations:** 1 Department of Oncology, Wayne State University School of Medicine, Detroit, MI USA; 2 Karmanos Cancer Institute, Wayne State University School of Medicine, Detroit, MI USA; 3 Department of Chemistry and Biochemistry, University of Michigan-Flint, Flint, MI USA

## Abstract

Embryonic stem (ES) cell pluripotency is governed by OCT4-centric transcriptional networks. Conventional ES cells can be derived and maintained *in vitro* with media containing the cytokine leukemia inhibitory factor (LIF), which propagates the pluripotent state by activating STAT3 signaling, and simultaneous inhibition of glycogen synthase kinase-3 (GSK3) and MAP kinase/ERK kinase signaling. However, it is unclear whether overexpression of OCT4 is sufficient to overcome LIF-dependence. Here, we show that inducible expression of OCT4 (iOCT4) supports long-term LIF-independent self-renewal of ES cells cultured in media containing fetal bovine serum (FBS) and a glycogen synthase kinase-3 (GSK3) inhibitor, and in serum-free media. Global expression analysis revealed that LIF-independent iOCT4 ES cells and control ES cells exhibit similar transcriptional programs relative to epiblast stem cells (EpiSCs) and differentiated cells. Epigenomic profiling also demonstrated similar patterns of histone modifications between LIF-independent iOCT4 and control ES cells. Moreover, LIF-independent iOCT4 ES cells retain the capacity to differentiate *in vitro* and *in vivo* upon downregulation of OCT4 expression. These findings indicate that OCT4 expression is sufficient to sustain intrinsic signaling in a LIF-independent manner to promote ES cell pluripotency and self-renewal.

## Introduction

Pluripotent embryonic stem (ES) cells derived from the inner cell mass of mouse preimplantation-stage embryos retain the capacity to self-renewal *in vitro* indefinitely^1,2^ in the presence of external stimuli such as leukemia inhibitory factor (LIF) and BMP4 or serum^3^. The POU class 5 transcription factor (Pou5f1) OCT4 is highly expressed in the inner cell mass (ICM) of blastocyst-stage embryos and is critical for maintaining the pluripotent state of ES cells^4,5^. Downregulation^5^ or deletion^6^ of OCT4 in ES cells leads to trophectodermal differentiation whereas upregulation of OCT4 leads to primitive endoderm and mesodermal differentiation^5^. The expression level of OCT4 is presumed to balance self-renewal and differentiation by activating or repressing transcription^7^. OCT4 is thought to promote self-renewal by establishing a cis-regulatory network with SOX2 and other key regulatory factors to co-bind multiple genes^8,9^. ES cell fate decisions are largely dictated by the interplay between external signaling pathways and intrinsic transcriptional networks^9^. ES cell self-renewal can be propagated without STAT3 activation, albeit with decreased quality, by inhibiting ERK signaling^10^ or by forced expression of NANOG^11^, KLF2^12^, KLF4, TBX3^13^, ESRRB^14^, GBX2^15^, and Tfcp2l1^16^. While these studies demonstrate that OCT4 is a critical regulator of ES cell self-renewal, it is unclear whether expression of OCT4 is sufficient to propagate ES cells in the absence of LIF.

Here, we investigated whether expression of OCT4 supports LIF-independent culture of ES cells. We demonstrate that exogenous OCT4 expression in combination with a wild-type endogenous OCT4 allele is sufficient to sustain self-renewal of ES cells cultured in media with or without FBS or GSK3i, and in the absence of LIF. While LIF-independent iOCT4 ES cells and wild-type ES cells exhibit overall similar transcriptional programs relative to epiblast stem cells (EpiSCs) and differentiated cells, global expression analysis demonstrated that a subset of STAT3 targets are downregulated in LIF-independent ES cells, while a subset of OCT4/STAT3 co-bound targets are upregulated. These results suggest that OCT4 may promote self-renewal in the absence of LIF/STAT3 signaling by driving expression of genes essential for maintaining pluripotency. The convergence of transcriptional networks between wild-type and LIF-independent ES cells may represent a minimal ground state network required for ES cell pluripotency. Epigenomic analyses also revealed similar patterns of histone modifications between LIF-independent iOCT4 and wild-type ES cells. Moreover, LIF-independent iOCT4 ES cells retain the capacity to differentiate *in vitro* and *in vivo* upon downregulation of OCT4 expression. These findings indicate that OCT4 expression is sufficient to sustain intrinsic signaling in a LIF-independent manner to promote ES cell pluripotency and self-renewal.

## Results

**Figure 1:**
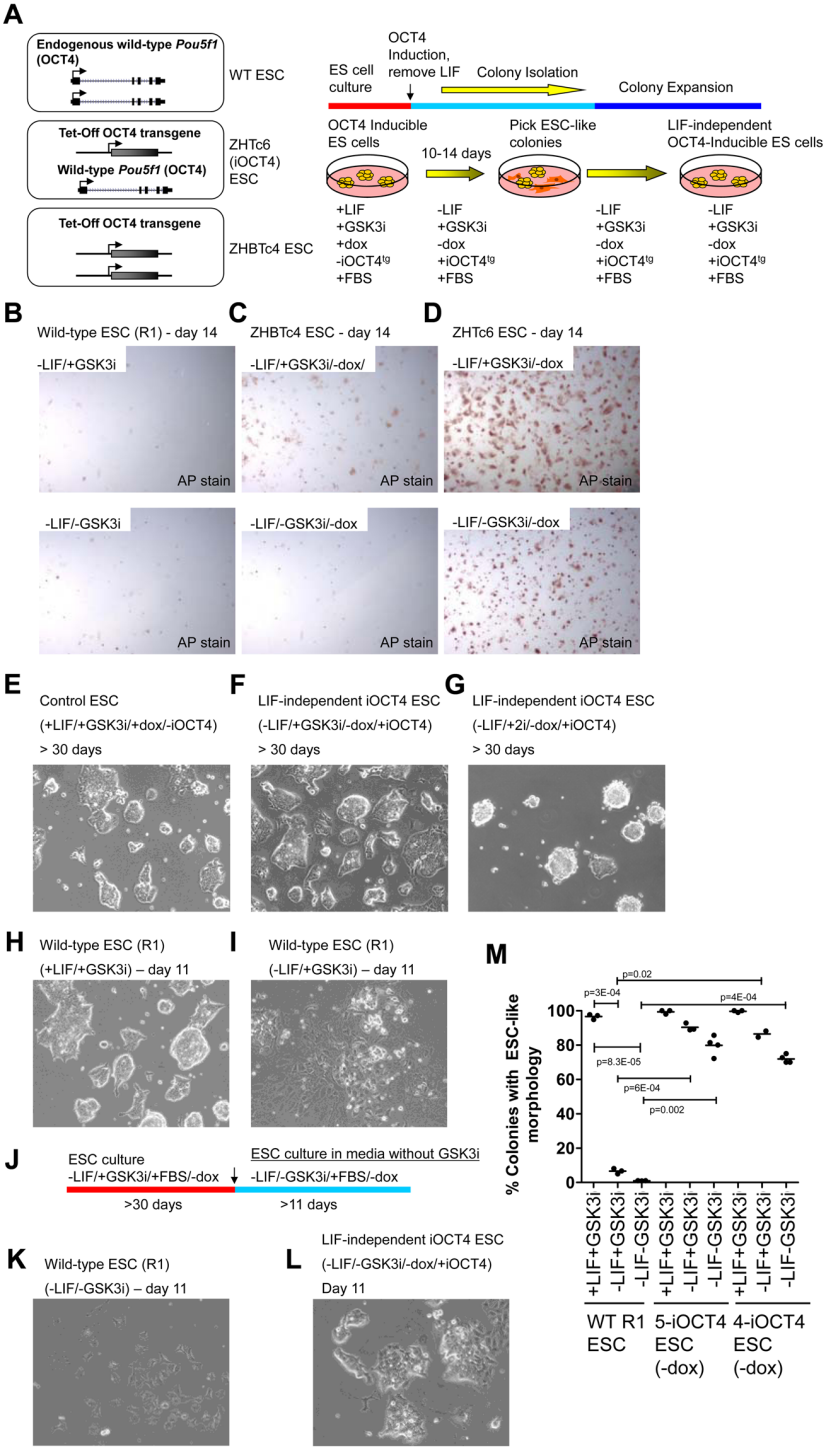
OCT4 overexpression sustains ES cell self-renewal in the absence of LIF (**A**) Schematic of endogenous wild-type *Pou5f1* (OCT4) alleles and the Tet-Off OCT4 transgene (left). Wild-type ES cells possess two endogenous wild-type *Pou5f1* (OCT4) alleles, ZHTc6 (OCT4-inducible; iOCT4) ES cells contain one wild-type OCT4 allele and one Tet-Off OCT4 transgene, and ZHBTc4 ES cells contain two Tet-Off OCT4 transgenes. The Tet-Off OCT4 transgene is activated in the absence of doxycycline. Schematic of experimental design (right). ZHTc6 OCT4-inducible (iOCT4) ES cells^5^ were grown in ES cell media containing fetal bovine serum (FBS) but without LIF, with GSK3i and without doxycycline to induce OCT4 transgene overexpression (tg; transgenic). ESC-like colonies were picked and expanded in LIF-independent ES cell media. (**B**) AP staining of wild-type ES cells cultured in FBS-containing media, and with GSK3i (top) or without GSK3i (bottom). (**C,D**) AP staining of LIF-independent iOCT4 ES cells cultured in LIF-independent ES cell media containing FBS with GSK3i (top) or without GSK3i (bottom). (**E–G**) Bright-field microscopy of (**E**) control (ZHTc6) and (**F,G**) LIF-independent iOCT4 ES cells cultured in the presence of (**E**) LIF and GSK3i, (**F**) GSK3i or (**G**) GSK3i/MEKi. Wild-type ES cells (R1) cultured in (**H**) ES cell media containing FBS and LIF (+LIF/+GSK3i) or (**I**) without LIF (−LIF/+GSK3i). (**J**) Schematic of experimental design. iOCT4 ES cells were grown in LIF-independent ES cell media containing FBS and without doxycycline (−LIF/+GSK3i/+FBS/−dox) to induce OCT4 transgene overexpression for at least 30 days. iOCT4 and wild-type ES cells were subsequently cultured in LIF-independent ES cell media without LIF, GSK3i, or doxycycline (−LIF/−GSK3i/+FBS/−dox) for 11 days. (**K**) Wild-type ES cells cultured in FBS-containing media but without LIF or GSK3i. (**L**) iOCT4 ES cells cultured in FBS-containing media but without LIF or GSK3i, and without doxycycline. (**M**) ES cell colonies were scored by morphology. The percentage of colonies with an ES-like morphology (compact and round vs. flattened) are represented as mean ± SEM. P values were calculated using a t test.

To investigate whether OCT4 expression is sufficient to propagate mouse ES cells in the absence of LIF we utilized the OCT4-regulatable ES cell line ZHTc6^5^. ZHTc6 ES cells have one allele inactivated by integration of an IRESzeopA cassette and contain a Tet-off OCT4 transgene^5^ (Fig. 1A, left). OCT4 transgene expression is activated in the absence of doxycycline. Under typical ES cell culture conditions in the presence of LIF, and with doxycycline to suppress OCT4 transgene expression, ZHTc6 ES cells exhibit normal self-renewal (Fig. 1A, right; E). In the presence of doxycycline and absence of LIF, ZHTc6 ES cells undergo differentiation^5^. To evaluate whether OCT4 expression is capable of sustaining ES cell self-renewal in the absence of LIF, we cultured OCT4 transgene inducible ZHTc6 (iOCT4) ES cells in the absence of LIF and doxycycline, and with or without inhibition of glycogen synthase kinase-3 (GSK3) (CHIR99021; GSK3i) (Fig. 1A, right). Previous results demonstrated that while constitutive activation of beta-catenin alone is unable to maintain self-renewal, GSK3i exhibits a synergistic effect with LIF^17^. This approach resulted in a mixed population of ESC-like colonies and differentiated cells over a time-course of two weeks. While many ZHTc6 (iOCT4) ES cell colonies expressed alkaline phosphatase (AP) when cultured in the absence of LIF, and with or without GSK3i (Fig. 1D), AP staining was largely absent following culture of wild-type ES cells in the absence of LIF, and with or without GSK3i (Fig. 1B). In addition, ZHBTc4 ES cells, which lack endogenous wild-type OCT4 expression and express two transgene-derived transcripts^5^ (Fig. 1A, left), also exhibited low levels of AP staining following culture of wild-type ES cells in the absence of LIF, and with or without GSK3i (Fig. 1C). This finding is in alignment with previous results, which showed that ZHBTc4 ES cells undergo differentiation when cultured in the absence of doxycycline and LIF^5^. Altogether, these results suggest that the combination of exogenous transgene and endogenous OCT4 expression in ZHTc6 ES cells is sufficient to maintain self-renewal in the absence of LIF, while exogenous OCT4 transgene expression alone in ZHBTc4 ES cells is insufficient to support self-renewal in the absence of LIF. Our results also suggest that GSK3i is not required to maintain self-renewal of ZHTc6 ES cells in the absence of LIF.

To characterize self-renewal of ZHTc6-derived (iOCT4) LIF-independent ES cells, eleven individual colonies were picked and expanded, and all of the clones were capable of self-renewing for >30 days without showing signs of decreased self-renewal. Two representative LIF-independent iOCT4 ES cell clones were subsequently evaluated further. The morphology of control ES cells (Fig. 1E) cultured in the presence of LIF and GSK3i was similar to a representative LIF-independent iOCT4 ES cell line cultured in ES cell media without LIF but containing GSK3i (Fig. 1F), or simultaneous inhibition of GSK3 and MAP kinase/ERK kinase signaling (2i) (Fig. 1G). In contrast, while wild-type ES cells cultured in ES cell media with LIF and GSK3i exhibit a normal ES cell morphology (Fig. 1H), wild-type ES cells cultured with GSK3i and without LIF became flattened and scattered, indicating a loss of self-renewal (Fig. 1I).

To determine whether GSK3i can promote ES cell self-renewal in the absence of LIF, we cultured wild-type and LIF-independent iOCT4 ES cells without GSK3i or LIF in ES cell media containing FBS (-LIF/-GSK3i) for at least four passages over a time-course of 11 days (Fig. 1J). While wild-type ES cell colonies became flattened and scattered, which is indicative of differentiation (Fig. 1K), the majority of LIF-independent iOCT4 ES cells maintained a 3D ESC-like colony morphology (Fig. 1L,M). These findings suggest that OCT4 is sufficient to promote self-renewal of ES cells cultured in FBS-containing media without GSK3i (Fig. 1M).

**Figure 2:**
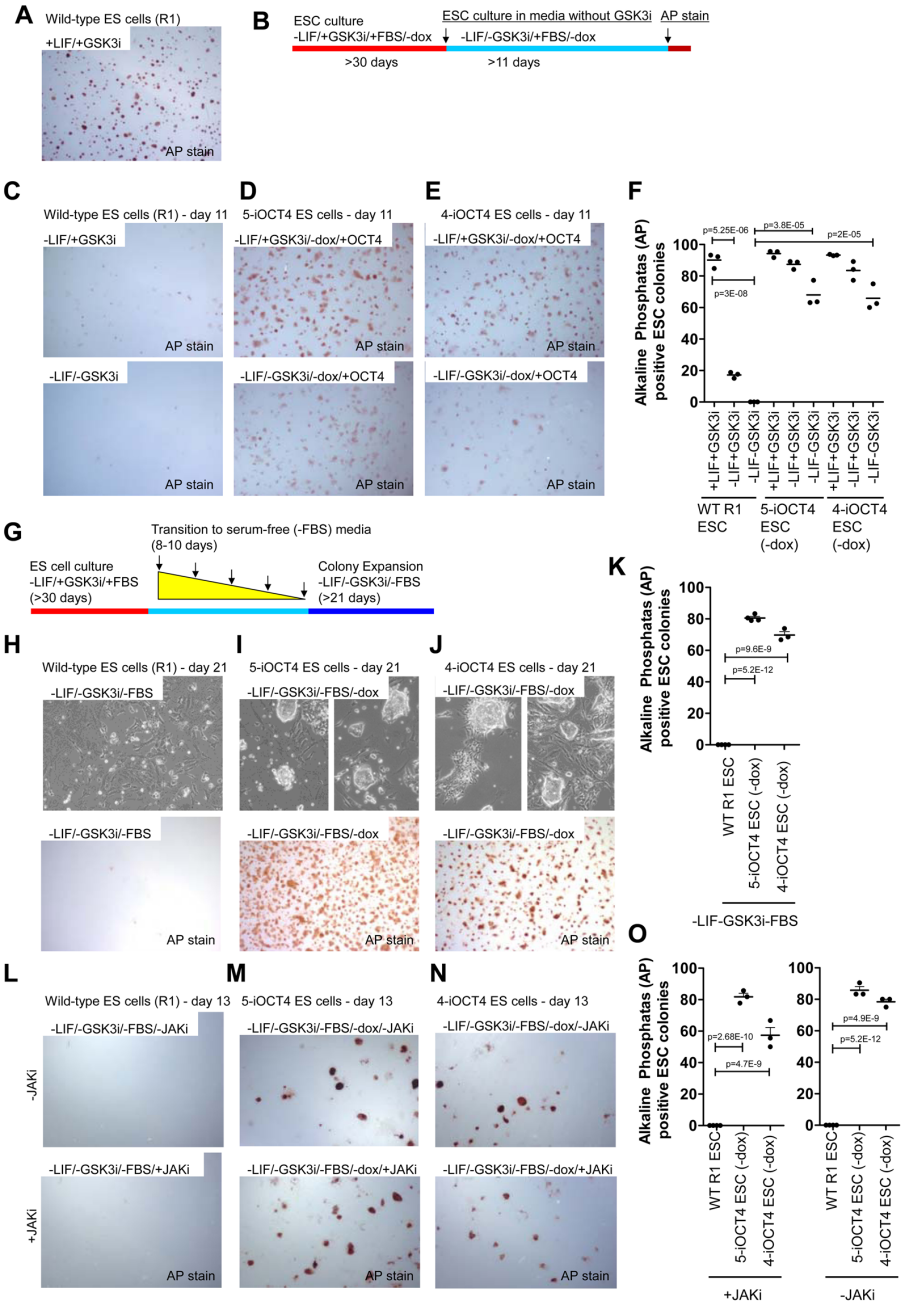
OCT4 overexpression promotes ES cell self-renewal without LIF, GSK3i, or FBS (**A**) Alkaline phosphatase (AP) staining of wild-type ES cells (R1) cultured in ES cell media containing FBS, LIF and GSK3i. (**B**) Schematic of experimental design. iOCT4 ES cells were grown in LIF-independent ES cell media containing FBS and without doxycycline (−LIF/+GSK3i/+FBS/−dox) to induce OCT4 transgene overexpression for at least 30 days. iOCT4 and wild-type ES cells were then cultured in LIF-independent ES cell media without LIF, GSK3i, or doxycycline (−LIF/−GSK3i/+FBS/−dox) for 11 days, and subsequently stained for AP. (**C**) AP staining of wild-type ES cells cultured in FBS-containing media, and with GSK3i (top) or without GSK3i (bottom). (**D,E**) AP staining of LIF-independent iOCT4 ES cells cultured in LIF-independent ES cell media containing FBS with GSK3i (top) or without GSK3i (bottom). (**F**) ES cells were scored by AP staining. The percentage of AP positive colonies is represented as mean ± SEM. p values were calculated using a t test. (**G**) Schematic of experimental design. Wild-type and iOCT4 ES cells were transitioned to serum-free media over 8–10 days, and subsequently cultured and passaged in serum-free media for 1–2 weeks. (**H**) Bright-field microscopy (top) and AP staining (bottom) of wild-type ES cells cultured in LIF-independent serum-free ES cell media without GSK3i for 21 days. (**I,J**) Bright-field microscopy (top) and AP staining (bottom) of iOCT4 ES cells cultured in LIF-independent serum-free ES cell media without GSK3i or doxycycline for 21 days. (**K**) ES cells were scored by AP staining. The percentage of AP positive colonies is represented as mean ± SEM. p values were calculated using a t test. (**L,N**) AP staining of (**L**) wild-type ES cells and (**M,N**) LIF-independent ES cells cultured at low density (500 cells / 6-well) in serum-free media, and with JAKi (bottom) or without JAKi (top). (**O**) ES cells were scored by AP staining. The percentage of AP positive colonies is represented as mean ± SEM. p values were calculated using a t test.

While most wild-type ES cell colonies expressed alkaline phosphatase (AP) when cultured in ES cell media containing LIF and GSK3i (Fig. 2A,F), the majority of wild-type ES cells cultured in the absence of LIF, and in the presence or absence of GSK3i, lacked AP staining (Fig. 2B,C,F). Moreover, none of the wild-type ES cell colonies exhibited an ESC-like morphology. In contrast, the majority of LIF-independent iOCT4 ES cells expressed AP when cultured in the absence of LIF and presence of GSK3i (Fig. 2D,E, top panels). Moreover, the majority of LIF-independent iOCT4 ES cells cultured without LIF or GSK3i were AP positive (Fig. 2D,E, bottom panels). In contrast, very few AP positive colonies were observed for wild-type ES cells cultured under the same conditions (Fig. 2F). Overall, these results suggest that induced exogenous OCT4 expression in combination with a wild-type endogenous OCT4 allele is sufficient to sustain self-renewal of ES cells cultured in FBS-containing media without LIF or GSK3i.

To rule out the possibility that spontaneously differentiated LIF-independent iOCT4 ES cells may produce LIF, which may support self-renewal of surrounding cells, we cultured LIF-independent iOCT4 ES cells at low density (500 cells/6-well; see methods) in FBS-containing ES cell media with or without a JAK kinase inhibitor (JAKi), which blocks STAT3 phosphorylation^18^. After culture for 12 days, wild-type ES cells were devoid of AP staining (Supplemental Fig. 1A). In addition, while we observed a slight decrease in the percentage of AP positive LIF-independent iOCT4 ES cell colonies that were cultured in the presence of JAKi relative to cells cultured without JAKi (Supplemental Fig. 1B–D), inhibition of JAK kinase did not ablate self-renewal.

To determine whether LIF-independent iOCT4 ES cells are capable of self-renewing in the absence of FBS, LIF, and GSK3i, we transitioned LIF-independent iOCT4 ES cells from ES cell media containing FBS to defined serum-free (SF) ES cell media over a time course of 8–10 days (Fig. 2G). Wild-type and iOCT4 ES cells were subsequently cultured in defined serum-free ES cell media without LIF or GSK3i for at least 21 days. Following their culture in serum-free ES cell media for 7 days, wild-type ES cells became flattened and scattered (Supplemental Fig. 1E, top panel), and lost AP staining (Supplemental Fig. 1E, bottom panel), demonstrating their inability to self-renew under these conditions. However, >50% of LIF-independent iOCT4 ES cell colonies were AP positive and maintained an ESC-like morphology when cultured in SF media without LIF or GSK3i for 7 days (Supplemental Fig. 1F–H). After culture for an extended period of time (21 days), wild-type ES cell colonies remained flattened and scattered (Fig. 2H,K) and were devoid of AP staining (Fig. 2H, bottom panel), while >70% of LIF-independent iOCT4 ES cell colonies were AP positive and maintained an ESC-like morphology (Fig. 2I–K). Combined, these results suggest that OCT4 expression is sufficient to sustain self-renewal of ES cells in the absence of LIF, GSK3i, and FBS.

To rule out the possibility that spontaneously differentiated LIF-independent iOCT4 ES cells may produce LIF, which may support self-renewal of surrounding cells, we cultured serum-free transitioned LIF-independent iOCT4 ES cells at low density (500 cells/6-well; see methods) in serum-free media with or without a JAK kinase inhibitor (JAKi) in a similar manner as described above. After culture for 13 days, wild-type ES cells were devoid of AP staining (Fig. 2L). In addition, while we observed a slight decrease in the percentage of AP positive LIF-independent iOCT4 ES cell colonies that were cultured in the presence JAKi relative to cells cultured without JAKi (Fig. 2M–O), inhibition of JAK kinase did not ablate self-renewal. We also observed similar findings for LIF-independent iOCT4 ES cells cultured at a moderate cell density (2,500 cells/6-well) in serum-free media with or without JAKi. After 10 days, while wild-type ES cells were devoid of AP staining (Supplemental Fig. 1I), many LIF-independent iOCT4 ES cells were AP positive (Supplemental Fig. 1J,K), suggesting that treatment of LIF-independent iOCT4 ES cells with JAKi does not ablate self-renewal. Taken together, these results demonstrate that the LIF-independent self-renewal characteristics of iOCT4 ES cells is not dependent on LIF produced from surrounding differentiated cells.

**Figure 3:**
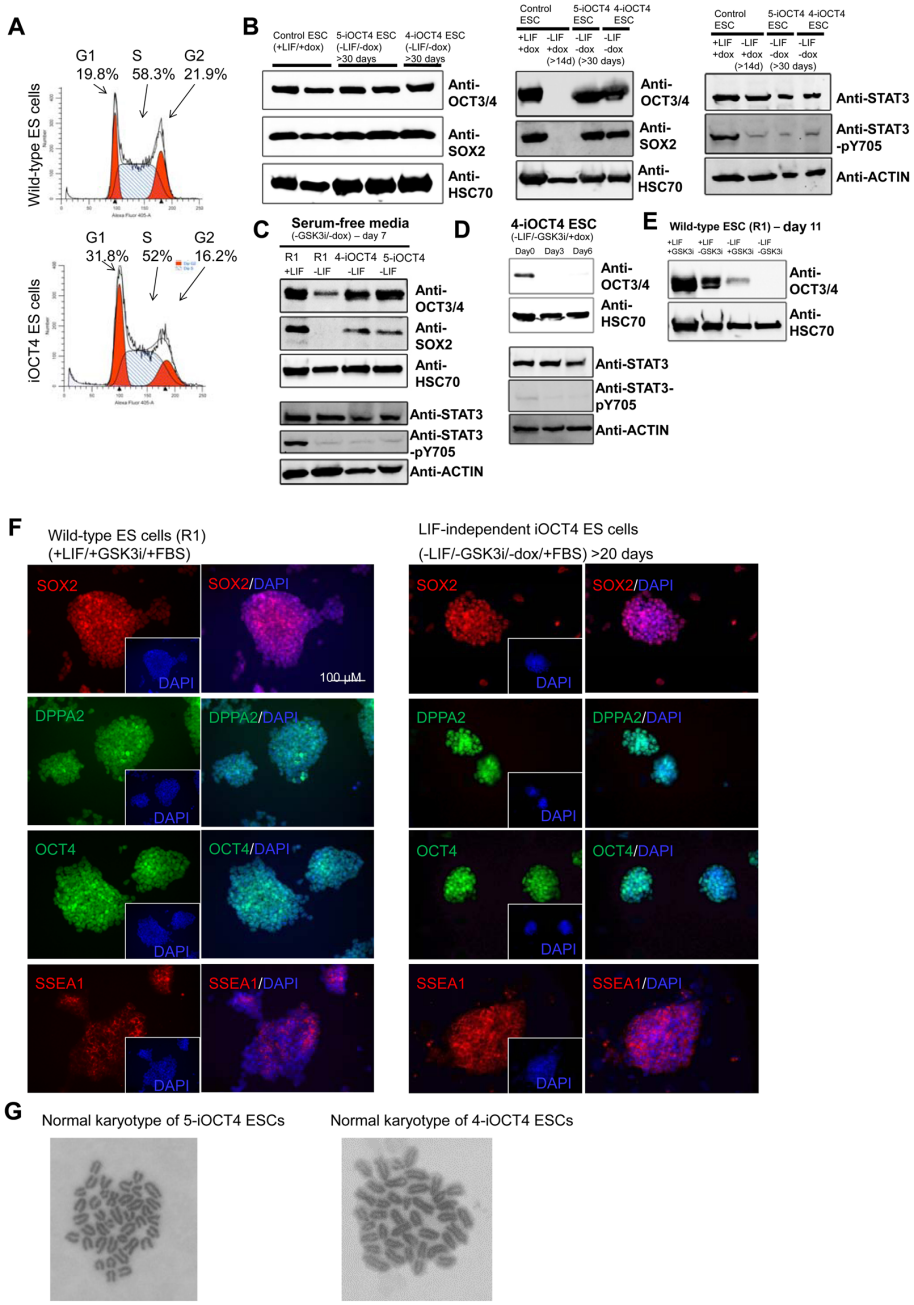
Characterization of iOCT4 ES cells cultured in LIF-independent ES cell media (**A**) Dapi cell cycle staining of wild-type ES cells (+LIF/+GSK3i) and iOCT4 LIF-independent iOCT4 ES cells (−LIF/+GSK3i/−dox) cultured in FBS-containing ES cell media. (**B**) Western blot of OCT4 and SOX2 (left and middle), and STAT3 and STAT3-pY705 (right), in control (ZHTc6) ES cells and LIF-independent iOCT4 ES cells cultured in FBS-containing media and GSK3i. HSC70 or ACTIN were used as loading controls. (**C**) Western blot of OCT4 and SOX2 (top), and STAT3 and STAT3-pY705 (bottom), in wild-type (R1) and LIF-independent iOCT4 ES cells cultured in serum-free ES cell media without GSK3i or doxycycline. (**D**) Western blot of OCT4 (top), and STAT3 and STAT3-pY705 (bottom), in LIF-independent iOCT4 ES cells cultured without GSK3i or LIF and with doxycycline to repress OCT4 transgene expression over a time-course of 6 days. Note that the level of OCT4 protein is significantly decreased after three days of culture in the presence of doxycycline. (**E**) Western blot of OCT4 in wild-type (R1) ES cells cultured in FBS-containing ES cell media with or without LIF, and with or without GSK3i for 11 days. Note that the level of OCT4 protein is significantly reduced in wild-type ES cells cultured in the absence of LIF. (**F**) Immunofluorescence analysis of SOX2, DPPA2, OCT4, and SSEA1 in control ES cells and LIF-independent iOCT4 ES cells cultured in FBS-containing ES cell media without LIF, GSK3i, or doxycycline. Nuclei were stained with Dapi. (**G**) Giesma staining of metaphase spreads and chromosome counting revealed that iOCT4 LIF-independent ES cells cultured in FBS-containing media and GSK3i have a normal karyotype.

However, we observed minor alterations in the cell cycle pattern of LIF-independent iOCT4 ES cells relative to wild-type ES cells (Fig. 3A). For example, 19.8% of wild-type ES cells and 31.8% of LIF-independent iOCT4 ES cells were in the G1 phase, 58.3% of wild-type and 52% of LIF-independent iOCT4 ES cells were in the S phase, and 21.9% and 16.2% were in the G2 phase of the cell cycle (Fig. 3A). These results suggest that LIF-independent iOCT4 ES cells exhibit a longer cell cycle relative to wild-type ES cells. These results may also indicate that LIF-independent iOCT4 ES cells exhibit decreased self-renewal relative to wild-type ES cells cultured in the presence of LIF. Western blot analysis demonstrated that OCT4 and SOX2 protein levels were similar between control ES cells (+LIF/+GSK3i/+dox/+FBS) and LIF-independent iOCT4 ES cells (−LIF/+GSK3i/−dox/+FBS) cultured for >30 days (Fig. 3B, Supplemental Fig. 2A, left). Moreover, OCT4 was also expressed in LIF-independent iOCT4 ES cells cultured in serum-free (SF) conditions and without GSK3i (−LIF/−GSK3i/−FBS) for 7 days (Fig. 3C, Supplemental Fig. 2B). In contrast, OCT4 and SOX2 levels were downregulated in wild-type ES cells cultured in serum-free and LIF-independent ES cell media for 7 days (Fig. 3C, Supplemental Fig. 2B). In addition, OCT4 levels were downregulated in LIF-independent iOCT4 ES cells in the absence of LIF and GSK3i and with the addition of doxycycline (Fig. 3D, Supplemental Fig. 2C). After three days, downregulation of OCT4 in the absence of LIF resulted in morphological changes indicative of differentiation, where ES cell colonies became flattened, lost their tight cell-cell contact, and became scattered at the colony periphery (Supplemental Fig. 2D). In contrast to iOCT4 ES cells, wild-type ES cells exhibited decreased levels of OCT4 in the absence of LIF and in the presence or absence of GSK3i (Fig. 3E, Supplemental Fig. 2E).

Because LIF signaling in mouse ES cells maintains pluripotency by inducing JAK-mediated phosphorylation of STAT3 Y705 (pY705)^19^, to rule out the effect of nuclear STAT3-pY705 in OCT4-dependent LIF-independent self-renewal, we evaluated STAT3-pY705 and total STAT3 levels in control and LIF-independent iOCT4 ES cells. Western blot analysis showed that while the level of STAT3-pY705 was high in control ES cells cultured in the presence of LIF and doxycycline (+LIF/+GSK3i/+dox/+FBS) (Fig. 3B, **right**, Supplemental Fig. 2A), the level of STAT3-pY705 was low in control ES cells cultured without LIF, and in LIF-independent iOCT4 ES cells (−LIF/+GSK3i/−dox/+FBS) cultured for >30 days (Fig. 3B, right, Supplemental Fig. 2A). In addition, STAT3-pY705 levels were low in LIF-independent iOCT4 ES cells cultured in serum-free (SF) conditions and without GSK3i (−LIF/−GSK3i/−dox/−FBS) for 7 days relative to wild-type ES cells cultured in the presence of LIF (Fig. 3C, bottom, Supplemental Fig. 2B). STAT3-pY705 levels also remained low in LIF-independent iOCT4 ES cells in the absence of LIF and GSK3i and with the addition of doxycycline (Fig. 3D, bottom, Supplemental Fig. 2C). These results demonstrate that self-renewal of LIF-independent iOCT4 ES cells is not dependent on constitutively activated STAT3 (STAT3-pY705).

Immunofluorescence staining confirmed that LIF-independent iOCT4 ES cells express the pluripotency markers SOX2, DPPA2, OCT4, and SSEA1 when cultured in absence of LIF, GSK3i, and doxycycline (Fig. 3F). Karyotyping analyses demonstrated that 5-iOCT4 and 4-iOCT4 ES cells exhibit a normal karyotype (40 chromosomes) (Fig. 3G). Overall, these results demonstrate that induced OCT4 expression is sufficient to sustain self-renewal of ES cells in the absence of LIF.

### LIF-independent ES cells exhibit a similar transcriptional profile relative to wild-type ES cells

**Figure 4:**
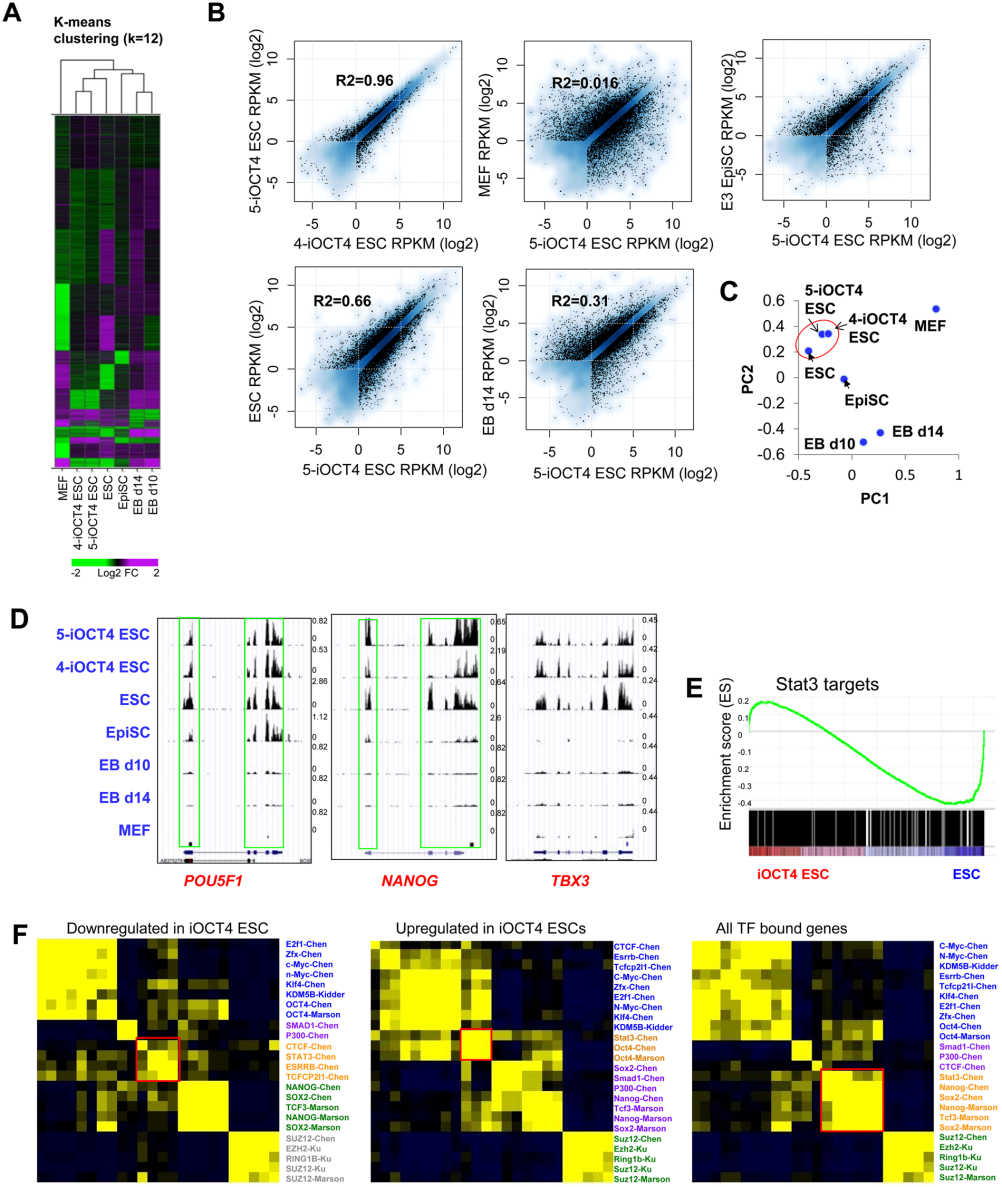
Expression profiling of LIF-independent iOCT4 ES cells (**A**) K-means clustering analysis of RNA-Seq data. Differentially expressed genes (> two-fold) clustered according to k-means. (**B**) Scatter plot of RNA-Seq gene expression analysis between two LIF-independent iOCT4 ES cell clones (#4 and #5) (top left), and between LIF-independent iOCT4 ES cells and control ES cells (bottom left), EpiSCs (top right), MEFs (top middle), or day 14 embryoid body (EB) differentiated ES cells (bottom right). Log2 adjusted differentially expressed genes are plotted (> two-fold, RPKM >1, FDR <0.001). (**C**) Principal component analysis (PCA) of differentially expressed genes between LIF-independent iOCT4 ES cells, control ES cells, EB-differentiated cells, EpiSCs, and MEFs. (**D**) Custom tracks of RNA-Seq data in the UCSC genome browser. (**E**) GSEA analysis of STAT3 targets between control ES cells and LIF-independent iOCT4 ES cells. (**F**) Correlation matrix of differentially expressed (DE) genes between control ES cells and LIF-independent iOCT4 ES cells with promoter binding of transcription factors and epigenetic modifiers. Heat map generated by evaluating pair-wise affinities between DE genes using RNA-Seq datasets generated from this study and published ChIP-Seq data^9,23–25^. AutoSOME^73^ was used to generate pair-wise affinity values.

To determine whether LIF-independent iOCT4 ES cells retain an ESC-like endogenous transcriptional circuitry we performed RNA-Seq analysis. The transcriptomes of two LIF-independent iOCT4 ES cell lines cultured in serum-containing media (−LIF+GSK3i−dox+FBS) were evaluated relative to wild-type ES cells (+LIF+GSK3i+FBS), epiblast stem cells (EpiSCs), wild-type ES cells differentiated into embryoid bodies (EBs) for 10 and 14 days, and mouse embryonic fibroblasts (MEFs). Wild-type ES cells serve as a positive control while EBs and MEFs serve as a negative control. EpiSCs express OCT4 and retain pluripotent characteristics and therefore serve as an ‘intermediate’ control. Hierarchical clustering analysis (HCA) followed by k-means clustering (KMC) to further refine major patterns of gene expression variability demonstrated that LIF-independent iOCT4 ES cells and wild-type ES cells clustered closer together relative to EBs and MEF differentiated cells (Fig. 4A). LIF-independent iOCT4 ES cells and EpiSCs also shared similar expression patterns at a subset of genes (Fig. 4A). Moreover, scatter plots demonstrated that two LIF-independent iOCT4 ES cell lines exhibited highly similar expression programs (Fig. 4B), and expression profiles of LIF-independent iOCT4 ES cell lines were more similar to wild-type ES cells relative to differentiated cells (EBs and MEFs). Principal component analysis (PCA) confirmed these results: LIF-independent iOCT4 ES cells clustered closer to wild-type ES cells relative to EpiSCs, differentiated EBs, or MEFs (Fig. 4C). Visualization of RNA-Seq tracks on the UCSC genome browser demonstrated that LIF-independent iOCT4 ES cells and wild-type ES cells express the pluripotency-regulators *Pou5f1*, *Nanog*, and *Tbx3* (Fig. 4D), while EpiSCs only express *Pou5f1*, and EB day 10, EB day 14, and MEFs do not express any of these genes.

STAT3 signaling is essential for conventional ES cell culture models^20,21^. However, STAT3 null ES cells can be propagated in serum-free conditions in the presence of GSK3i/MEKi and FGF receptor tyrosine kinases (3i)^10^, albeit with decreased quality, suggesting that LIF-independent maintenance of ES cell self-renewal can be bypassed by activation of alternative or complementary pluripotency-networks. To investigate the expression state of STAT3 target genes in LIF-independent iOCT4 ES cells relative to wild-type ES cells we used gene set enrichment analysis (GSEA)^22^. These findings revealed that expression of STAT3 targets is higher in wild-type ES cells relative to LIF-independent iOCT4 ES cells (Fig. 4E), suggesting that LIF-independent iOCT4 ES cells utilize a non-LIF-STAT3-centered network to maintain self-renewal. To further investigate this phenomenon we evaluated whether promoter regions nearby differentially expressed genes between LIF-independent and wild-type ES cells are occupied by pluripotency-regulators or epigenetic modifiers using public datasets^9,23–25^. We observed a correlation between downregulated genes in LIF-independent iOCT4 ES cells and those bound by STAT3 without OCT4, and a correlation between upregulated genes in LIF-independent iOCT4 ES cells and those bound by OCT4/STAT3 (Fig. 4F). These findings suggest that STAT3-bound genes, which are downregulated in LIF-independent iOCT4 ES cells, may be dispensable for maintaining ES cell self-renewal. However, OCT4/STAT3 co-bound genes, which are upregulated in LIF-independent iOCT4 ES cells, may be critical regulators of ES cell self-renewal. Upregulation of OCT4/STAT3 co-bound genes may be due to the repressive function of STAT3, as STAT3 has previously been shown to act as a transcriptional activator or repressor in ES cells^26^. In conventional ES cell culture conditions containing LIF, STAT3 binding may partially repress the expression of OCT4 target genes, or cooperate with OCT4 to drive expression of pluripotency genes. Alternatively, upregulation of OCT4/STAT3 targets may be a result of OCT4 compensating for a lack of externally activated LIF-STAT3 signaling by driving an intrinsic OCT4-centered transcriptional network.

Because transcriptome analyses were performed using wild-type and LIF-independent iOCT4 ES cells that were cultured in medium containing GSK3i, we investigated whether Wnt target genes are differentially expressed between LIF-independent iOCT4 ES cells and wild-type ES cells. Results from these analyses demonstrated that 12 Wnt target genes were upregulated, including Cdh1^27^, Cdkn2a^28^, and Id2^29^, while 12 Wnt target genes were downregulated, including Ccnd1^30^, Eno1^31^, Fgf4^32^, Gbx2^33^, Fzd7^34^, Myc^35^, and Sfrp1^36^, in LIF-independent iOCT4 ES cells relative to control ES cells. Because we observed differential expression of 24 Wnt target genes between LIF-independent iOCT4 ES cells and wild-type ES cells, we cannot rule out the possibility that changes in expression of direct or indirect Wnt target genes may be due in part to the presence of GSK3i in the culture media.

### Comparable epigenetic landscape of LIF-independent and wild-type ES cells

**Figure 5:**
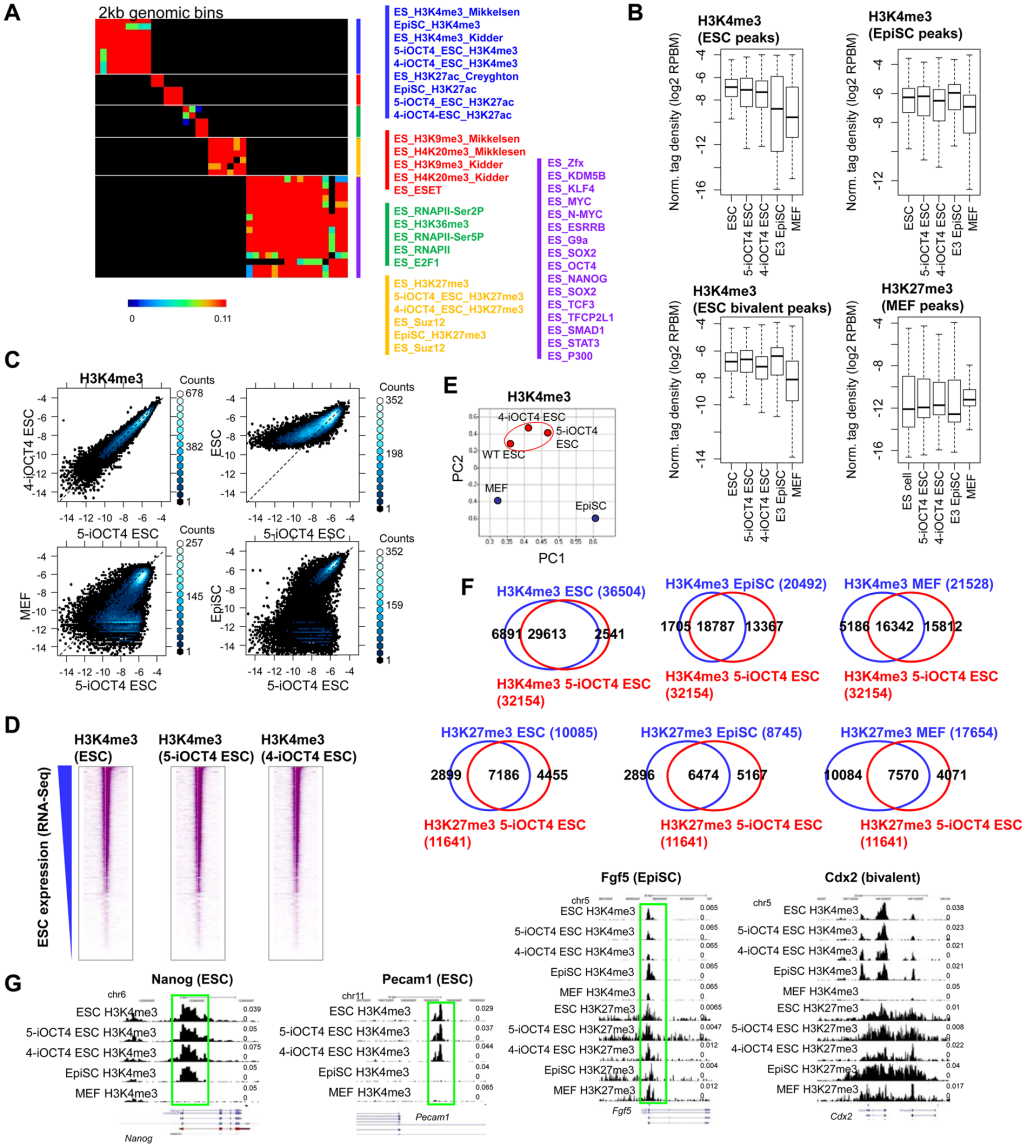
Epigenetic profiling of LIF-independent iOCT4 ES cells (**A**) Correlation matrix of densities of histone modifications, transcriptional regulators, and epigenetic regulators at 2 kb genomic intervals between control ES cells, LIF-independent iOCT4 ES cells, and EpiSCs. Heat map generated by evaluating pair-wise affinities between DE genes using RNA-Seq datasets generated from this study and published ChIP-Seq data^9,23–25^. AutoSOME^73^ was used to generate pair-wise affinity values. (**B**) Boxplot of H3K4me3 densities at ESC-peaks and H3K27me3-densities at MEF-peaks in control ES cells, LIF-independent iOCT4 ES cells, EpiSCs, and MEFs. (**C**) Scatter plots (log2 normalized tag density; RPBM) and (**D**) heat maps of H3K4me3 densities at ES cell ChIP-enriched peaks. (**E**) Principal component analysis (PCA) of H3K4me3 levels between LIF-independent iOCT4 ES cells, wild-type ES cells, EpiSCs, and MEFs. (**F**) Venn diagrams showing overlap between histone modification ChIP-peaks in control ES cells, LIF-independent iOCT4 ES cells, EpiSCs, and MEFs. (**G**) Browser view of H3K4me3 and H3K27me3 in control ES cells, LIF-independent iOCT4 ES cells, EpiSCs, and MEFs.

To investigate the genome-wide distribution of histone modifications in LIF-independent iOCT4 ES cells cultured in serum-containing media (−LIF + GSK3i−dox + FBS), we performed ChIP-Seq using methods previously described^25,37,38^. We then compared LIF-independent ES cell histone modifications profiles with histone modification and transcription factor binding patterns in wild-type ES cells using 2 kb genomic bins (Fig. 5A). These results demonstrate that global histone modification profiles of LIF-independent iOCT4 ES cells are similar to wild-type ES cells. Boxplots also show that global levels of H3K4me3 at ES cell-enriched regions are similar between wild-type and LIF-independent iOCT4 ES cells (Fig. 5B, top left), while EpiSCs and MEFs (GSE21271) exhibited decreased levels at ESC SICER-defined peaks. Also, H3K4me3 levels at EpiSC-enriched regions were lower in wild-type and LIF-independent iOCT4 ES cells relative to EpiSCs (Fig. 5B, top right), suggesting that H3K4me3 levels in LIF-independent iOCT4 ES cells are more similar to wild-type ES cells and less similar to EpiSCs. Moreover, H3K4me3 levels at H3K4me3/H3K27me3 bivalently marked chromatin were similar between wild-type ES cells, LIF-independent iOCT4 ES cells, and EpiSCs (Fig. 5B, bottom left). Scatter plots confirmed that two LIF-independent ES cell lines exhibited similar H3K4me3 profiles (Fig. 5C). While LIF-independent iOCT4 ES cells were more similar to wild-type ES cells relative to EpiSCs or MEFs, a comparison of wild-type and LIF-independent iOCT4 ES cells revealed that H3K4me3 levels were lower at a subset of regions in LIF-independent iOCT4 ES cells. These results suggest that a subset of genes that are activated upon external activation of LIF-STAT3 signaling, and downregulated in LIF-independent iOCT4 ES cells, may be dispensable for pluripotency. Heat maps also showed that wild-type and LIF-independent iOCT4 ES cells exhibited similar genome-wide H3K4me3 levels (Fig. 5D). Principal component analysis (PCA) confirmed these results: LIF-independent iOCT4 ES cells clustered closer to wild-type ES cells relative to EpiSCs or MEFs (Fig. 5E). Moreover, our ChIP-Seq data demonstrate that 92% of H3K4me3-marked regions in LIF-independent iOCT4 ES cells were also marked by H3K4me3 in control ES cells (Fig. 5F, top left). In contrast, 58% and 51% of H3K4me3-marked regions in LIF-independent iOCT4 ES cells were also marked by H3K4me3 in EpiSCs and MEFs, respectively (Fig. 5F, top right). These results are similar to differences between wild-type ES cells and EpiSCs (59% of ESC H3K4me3-marked regions co-marked in EpiSCs). Moreover, 71% of H3K27me3-marked regions regions in wild-type ES cells were also marked by H3K27me3 in LIF-independent iOCT4 ES cells (Fig. 5F, bottom left). In contrast, 74% and 43% of H3K27me3-marked regions in EpiSCs and MEFs, respectively, were also marked by H3K27me3 in LIF-independent iOCT4 ES cells (Fig. 5F, bottom right). These slight differences in H3K27me3 levels between wild-type and LIF-independent iOCT4 ES cells may be attributed to cell culture conditions^39^, as wild-type ES cell H3K27me3 data was obtained from a public repository, while LIF-independent ES cell H3K27me3 data was generated for these studies. UCSC genome browser views revealed enrichment of H3K4me3 at pluripotency-genes such as *Nanog* (Fig. 5G, left) and *Pecam1* in LIF-independent iOCT4 ES cells, which is expressed in ES cells but not EpiSCs (Fig. 5G, left middle). In addition, while EpiSCs displayed elevated levels of H3K4me3 at *Fgf5* in EpiSCs, we observed low to moderate levels of H3K4me3 at the EpiSC marker *Fgf5* (Fig. 5G, right middle), further demonstrating that LIF-independent iOCT4 ES cells epigenetically resemble bona fide ES cells relative to epiblast-like stem cells. Furthermore, LIF-independent iOCT4 ES cells exhibit H3K4me3/H3K27me3 enrichment at a representative bivalently marked gene (Fig. 5G, right). Altogether, these results demonstrate the LIF-independent iOCT4 ES cells have a similar epigenetic profile relative to wild-type ES cells.

### Similar H3K27ac marking of active enhancers in LIF-independent and wild-type ES cells

**Figure 6:**
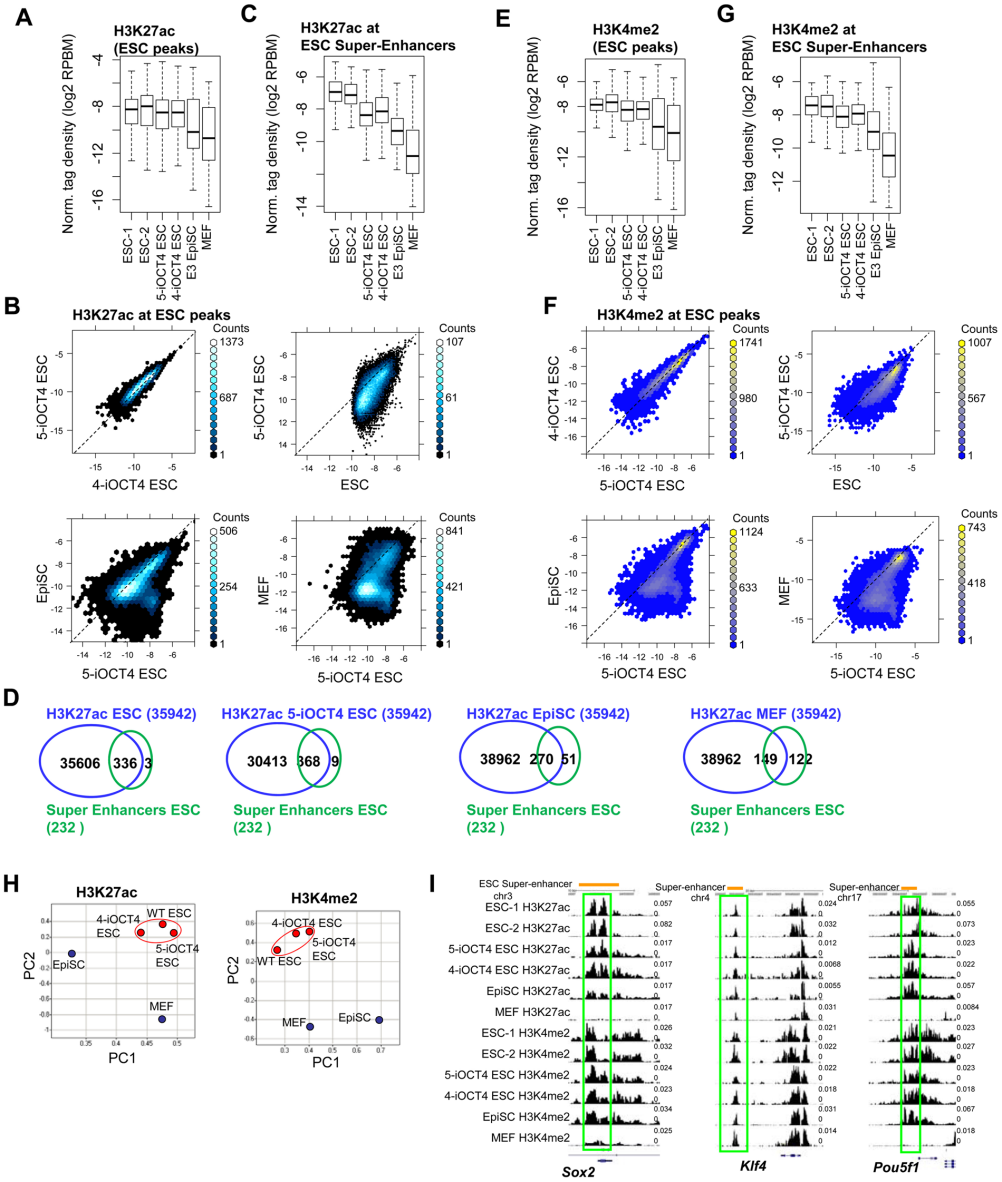
Enhancer profiling of LIF-independent iOCT4 ES cells (**A**) Boxplot and (**B**) scatter plots of H3K27ac densities (log2 normalized tag density; RPBM) at ESC-peaks in control ES cells, LIF-independent iOCT4 ES cells, EpiSCs, and MEFs. (**C**) Boxplot H3K27ac density at ESC super-enhancers. (**D**) Venn diagrams showing overlap between H3K27ac and ESC super-enhancers in control ES cells, LIF-independent iOCT4 ES cells, EpiSCs, and MEFs. (**E**) Boxplot and (**F**) scatter plots of H3K4me2 densities at ESC-peaks. (**G**) Boxplot of H3K4me2 density at ESC super-enhancers. (**H**) Principal component analysis (PCA) of H3K27ac and H3K4me2 levels between LIF-independent iOCT4 ES cells, wild-type ES cells, EpiSCs, and MEFs. (**I**) Browser view of H3K27ac and H3K4me2 in control ES cells, LIF-independent iOCT4 ES cells, EpiSCs, and MEFs.

*Cis-*regulatory elements are thought to regulate cell-type specific transcriptional events and responses to external stimuli^40–42^. To investigate whether enhancer profiles are similar between LIF-independent iOCT4 ES cells cultured in serum-containing media (−LIF + GSK3i−dox + FBS) and wild-type ES cells ( + LIF + GSK3i + FBS), we performed ChIP-Seq for H3K27ac, which marks active enhancers in intergenic regions^43,44^. We observed similar levels of intergenic H3K27ac levels between wild-type ES cells and two LIF-independent ES cell lines (Fig. 6A), while EpiSCs and MEFs (GSE29218) exhibited decreased levels at ESC-marked intergenic active enhancers, suggesting that H3K27ac-marked active enhancers in LIF-independent iOCT4 ES cells are more similar to wild-type ES cells than EpiSCs or MEFs. Furthermore, a direct comparison of two LIF-independent ES cell lines demonstrated that H3K27ac intergenic levels are highly similar (Fig. 6B, left panel). While H3K27ac levels were overall similar at intergenic regions in LIF-independent iOCT4 ES cells relative to wild-type ES cells (Fig. 6B, right panel), a subset of H3K27ac-marked enhancers displayed elevated levels in wild-type ES cells, suggesting that LIF-independent iOCT4 ES cells may have deactivated a subset of ESC-enhancers. Importantly, intergenic H3K27ac levels were different between LIF-independent iOCT4 ES cells and EpiSCs or MEFs (Fig. 6B, bottom). Recent work has demonstrated that master transcription factors and mediator co-bind clusters of enhancers, termed ‘super-enhancers’, which define cell identity^45,46^. To investigate whether ESC super-enhancers are active in LIF-independent iOCT4 ES cells, we evaluated the levels of H3K27ac at ESC-defined super-enhancers. Our findings demonstrate that H3K27ac levels are slightly lower in LIF-independent iOCT4 ES cells relative to wild-type ES cells (Fig. 6C). These findings are in alignment with decreased levels of H3K27ac at a subset of ESC-enhancers as described above, and likely reflect deactivation of enhancers that are dispensable for self-renewal in the absence of LIF. In addition, levels of H3K27ac at ESC-defined super-enhancers was higher in LIF-independent iOCT4 ES cells relative to EpiSCs or MEFs (Fig. 6C), demonstrating that ESC-defined super-enhancers are more active in LIF-independent iOCT4 ES cells relative to EpiSCs or MEFs. We also evaluated the number of H3K27ac ChIP peaks (SICER-defined peaks^47^) which overlap super-enhancer domains^45^. These results demonstrate that a similar number of H3K27ac-marked super-enhancers are active in LIF-independent and wild-type ES cells (Fig. 6D, left). In contrast, there were fewer H3K27ac-marked super-enhancers that were active in EpiSCs or MEFs (Fig. 6D, right), further demonstrating that LIF-independent iOCT4 ES cells are more similar to wild-type ES cells relative to EpiSCs or MEFs. It is noteworthy that the number of overlapping H3K27ac domains is greater than the total number of super-enhancers because super-enhancers are comprised of multiple H3K27ac domains.

To gain further insight into the enhancer landscape of LIF-independent iOCT4 ES cells relative to wild-type ES cells we surveyed H3K4me2 levels, which is another histone modification present at enhancer regions^48^. Like H3K27ac, H3K4me2 is also correlated with cell-type specific enhancer activity. To evaluate H3K4me2 levels we performed ChIP-Seq and observed similar levels of H3K4me2 between wild-type ES cells and two LIF-independent ES cell lines (Fig. 6E), while EpiSCs and MEFs (GSE36292)^49^ exhibited lower levels at ESC-marked H3K4me2 active enhancers, suggesting that LIF-independent iOCT4 ES cells are more similar to wild-type ES cells than EpiSCs or MEFs. Moreover, a comparison of two LIF-independent ES cell lines using scatter plots revealed highly similar levels of H3K4me2 (Fig. 6F, left). Likewise, H3K4me2 levels were similar between LIF-independent and wild-type ES cells (Fig. 6F, right). However, H3K4me2 levels were different between LIF-independent iOCT4 ES cells and EpiSCs or MEF (Fig. 6F, bottom), further suggesting that LIF-independent iOCT4 ES cells are more epigenetically similar to wild-type ES cells than EpiSCs or MEFs. Moreover, H3K4me2 levels at ESC-defined super-enhancers were similar, although slightly lower, relative to wild-type ES cells (Fig. 6G). However, H3K4me2 levels were higher at super-enhancers in LIF-independent iOCT4 ES cells relative to EpiSCs or MEFs (Fig. 6G). Furthermore, PCA showed that global levels of H3K27ac and H3K4me2 in LIF-independent iOCT4 ES cells clustered closer to wild-type ES cells relative to EpiSCs or MEFs (Fig. 6H). UCSC genome browser views confirm that H3K27ac and H3K4me2 levels at pluripotency-regulators in LIF-independent iOCT4 ES cells are more similar to wild-type ES cells and EpiSCs relative to MEFs (Fig. 6I). Overall, these suggest that the active enhancer profile of LIF-independent iOCT4 ES cells is similar to wild-type ES cells.

### LIF-independent iOCT4 ES cells are capable of differentiating in the absence of OCT4

**Figure 7:**
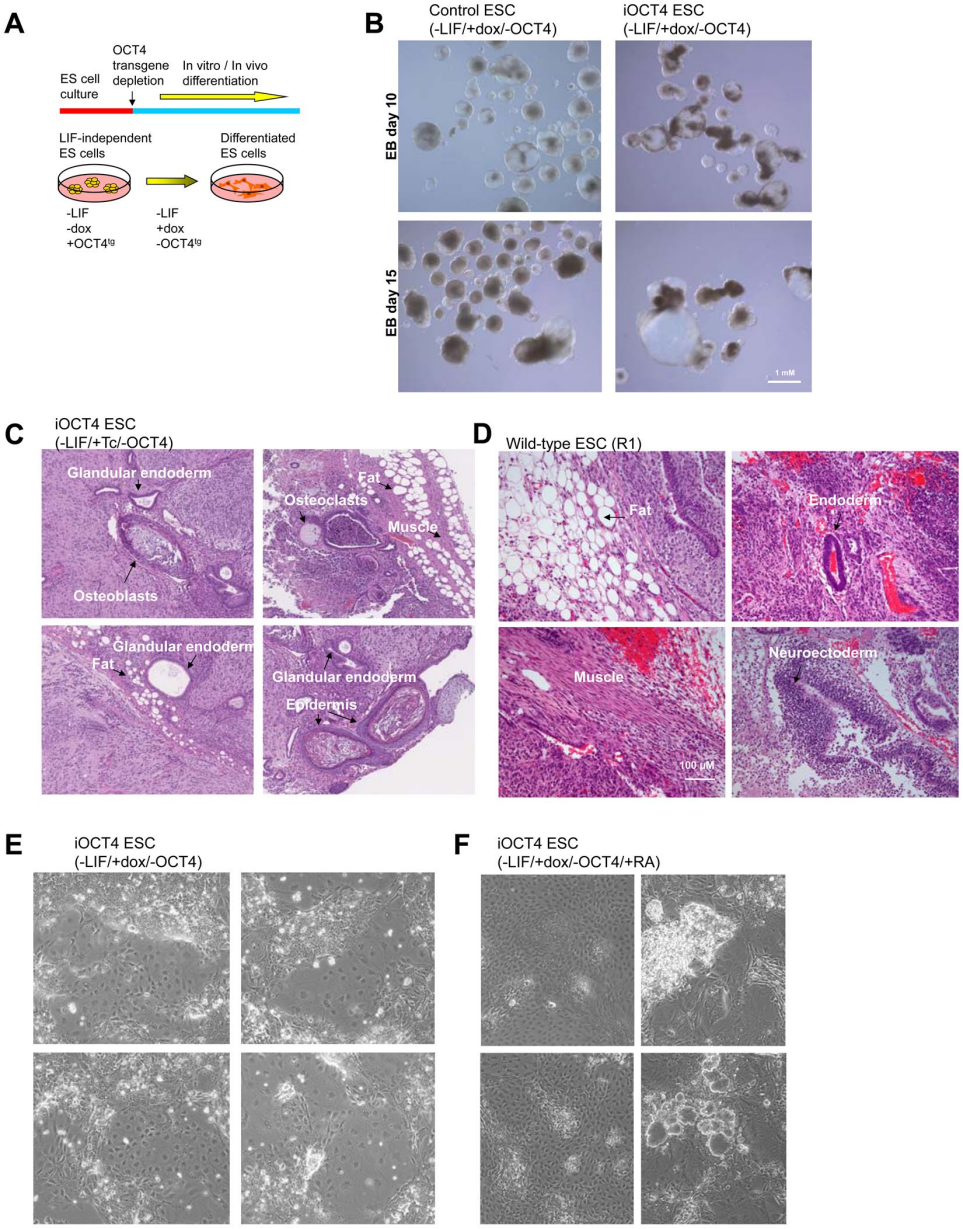
*In vitro* and *in vivo* differentiation potential of LIF-independent iOCT4 ES cells (**A**) Schematic of experimental design. To induce differentiation, OCT4-inducible (iOCT4) ES cells were grown in the absence of LIF and with doxycycline to downregulate OCT4 expression on low-binding dishes or as a monolayer. (**B**) Embryoid body (EB) differentiation of control ES cells (ZHTc6) and LIF-independent iOCT4 ES cells. (**C**) Teratomas generated from LIF-independent iOCT4 ES cells injected into SCID-beige mice, which were treated with tetracycline (Tc) to downregulate OCT4 expression. (**D**) Teratomas generated from wild-type ES cells (R1) injected into SCID-beige mice. Tumors were harvested 4–6 weeks post injection and evaluated using standard H&E histological methods. Transmitted white-light microscopy of sectioned teratomas. Heterogeneous differentiation of LIF-independent iOCT4 ES cells and control ES cells into endoderm (glandular structures), mesoderm (osteoblasts, adipocytes, muscle), and ectoderm (keratinized epidermal cells). (**E,F**) Differentiation of LIF-independent iOCT4 ES cells in the presence of doxycycline to downregulate OCT4 expression and (**E**) without or (**F**) with retinoic acid.

**Figure 8:**
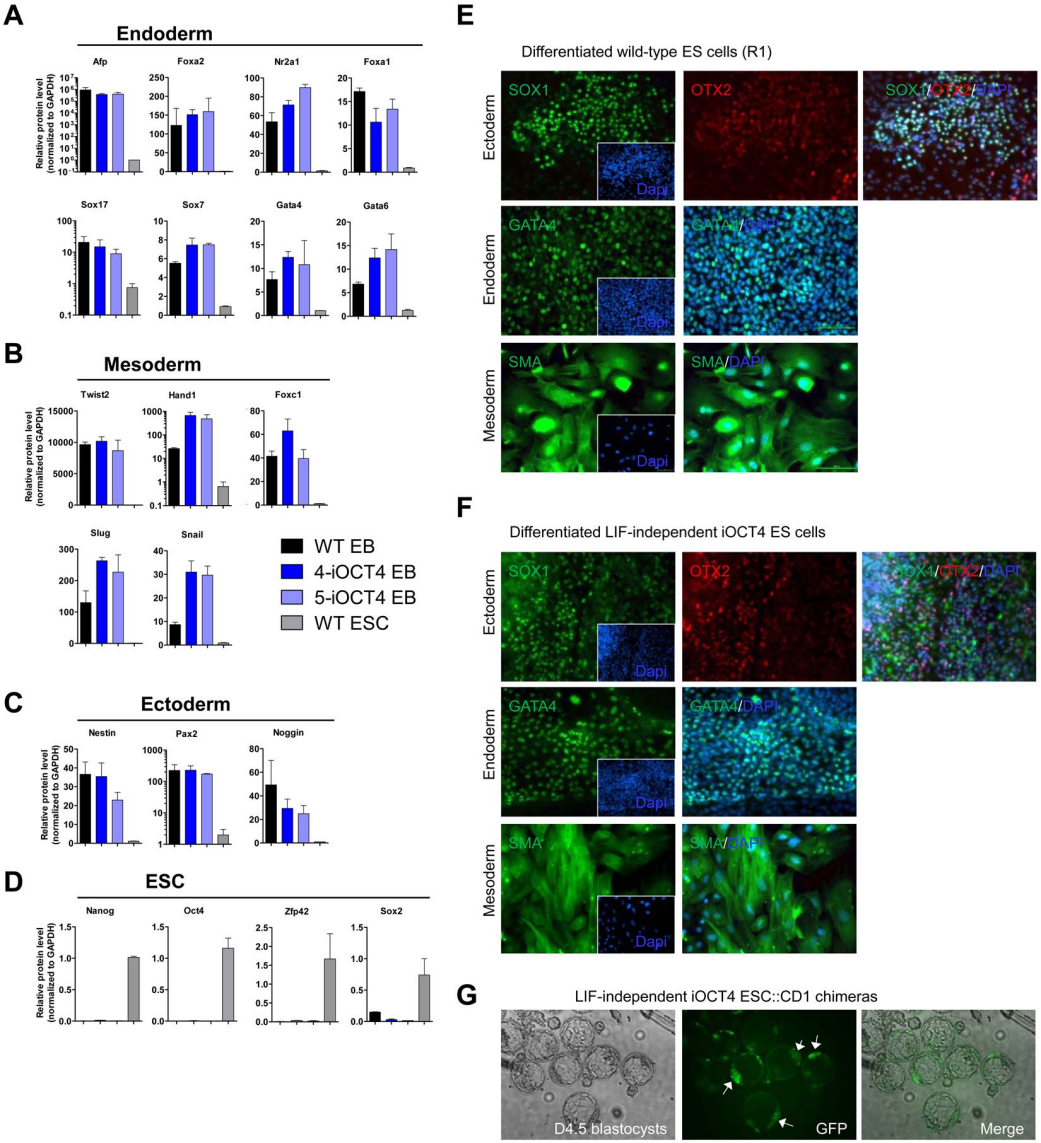
Differentiation of LIF-independent iOCT4 ES cells. Q-RT-PCR expression of (**A**) endoderm, (**B**) mesoderm, (**C**) ectoderm, and (**D**) ESC-genes in undifferentiated wild-type (R1) ES cells, and EB differentiated LIF-independent iOCT4 ES cells and wild-type (R1) ES cells for 14 days. mRNA levels are normalized to GAPDH and the expression of undifferentiated wild-type (R1) ES cells. (**E,F**) Immunofluorescence analysis of SOX1 and OTX2 (ectoderm), GATA4 (endoderm), and SMA (mesoderm) in (**E**) wild-type ES cells and (**F**) LIF-independent iOCT4 ES cells. Nuclei were stained with Dapi. (**G**) Day 4.5 blastocysts injected with LIF-independent iOCT4 ES cells (LIF-independent iOCT4 ES cell::CD1 chimeras). Arrows indicate location of inner cell mass (ICM).

To evaluate the ability of LIF-independent iOCT4 ES cells to differentiate we induced embryoid body (EB) formation following removal of LIF and addition of doxycycline in the culture media to downregulate OCT4 expression. LIF-independent iOCT4 ES cells were cultured without LIF and with doxycycline on low attachment dishes to induce EB differentiation over two weeks (Fig. 7A). LIF-independent and control ES cells formed cystic and globular EB structures containing a primitive endoderm layer (Fig. 7B). Differentiation potential of LIF-independent iOCT4 ES cells and wild-type ES cells was also evaluated using teratoma formation (Fig. 7C,D), where LIF-independent iOCT4 ES cells injected subcutaneously into SCID-beige immune-compromised mice, which were treated with tetracycline to downregulate OCT4 expression, differentiated into cells represented in the three germ layers including ectoderm (epidermis), mesoderm (osteoblasts, osteoclasts, fat, muscle), and endoderm (glandular endoderm) (Fig. 7C). Likewise, wild-type ES cells also differentiated into cells represented in the three germ layers including ectoderm (neuroectoderm), mesoderm (muscle, fat), and endoderm (glandular endoderm) (Fig. 7D). Moreover, LIF-independent iOCT4 ES cells differentiated as a monolayer on gelatin-coated dishes into a heterogeneous population of cells in the absence of LIF and with doxycycline (Fig. 7E), or in the absence of LIF and with doxycycline and retinoic acid (Fig. 7F). The ability to form EBs and generate teratomas upon downregulation of OCT4 demonstrates the functionality of LIF-independent iOCT4 ES cells. Moreover, we evaluated the expression state of lineage-specific genes following embryoid body induction of wild-type and LIF-independent iOCT4 ES cells in the absence of LIF and with doxycycline for 14 days, and found that lineage-specific genes, including Afp, Foxa2, Nr2a1, Foxa1, Sox7, Sox17, Gata4, Gata6 (endoderm; Fig. 8A), Twist2, Hand1, Foxc1, Slug, Snail (mesoderm; Fig. 8B), Nestin, Pax2, and Noggin (ectoderm; Fig. 8C), were upregulated in wild-type and LIF-independent iOCT4 EBs relative to undifferentiated ES cells. In addition, Nanog, Oct4, Sox2, and Zfp42 (ESC-genes; Fig. 8D) were downregulated in wild-type and LIF-independent iOCT4 EBs relative to undifferentiated ES cells. Moreover, immunofluorescence staining demonstrated that differentiated LIF-independent iOCT4 ES cells and wild-type ES cells express SOX1 and OTX2 (ectoderm), GATA4 (endoderm), and SMA (mesoderm) (Fig. 8E,F). We also observed spontaneously beating cardiomyocyte-like cells derived from wild-type (Supplemental Movie 1) and LIF-independent iOCT4 differentiated ES cells (Supplemental Movie 2). Combined, these results demonstrate that LIF-independent iOCT4 ES cells are capable of differentiating following downregulation of OCT4. In addition, to evaluate the potential of LIF-independent iOCT4 ES cells to home to the blastocyst inner cell mass (ICM), we transduced LIF-independent iOCT4 ES cells with lentiviral particles encoding GFP and subsequently injected them into E3.5 blastocyst-stage embryos. We then investigated the contribution of LIF-independent iOCT4 ES cells to mouse blastocyst chimeras following *in vitro* culture. After twenty-four hours we observed contribution to the blastocyst ICM (Fig. 8G). These results indicate that LIF-independent iOCT4 ES cells are capable of integrating into the ICM. Overall, our findings demonstrate that forced expression of OCT4 confers LIF-independent self-renewal of ES cells.

## Discussion

Integration of external LIF signaling through STAT3 activation is thought to work in concert with an intrinsic OCT4-centric transcriptional network to sustain ES cell self-renewal^9,20,50^. Previous studies suggest that OCT4 plays a central role in propagating ES cell self-renewal in differentiation conditions by inducing Klf2^12^, which is a downstream target of OCT4. These findings implicate a potential role for OCT4 in propagating ES cells in the absence of LIF. Also, it has been suggested that transient OCT4 expression can temporarily delay differentiation^51^; however, it has not been fully demonstrated that OCT4 can promote ES cell self-renewal independent of LIF indefinitely. Therefore, in this study we investigated whether expression of OCT4 supports long-term self-renewal of ES cells in the absence of LIF. Our findings demonstrate that forced expression of exogenous OCT4 in combination with a wild-type endogenous OCT4 allele is sufficient to sustain self-renewal in LIF-independent culture conditions. We further reveal that LIF-independent iOCT4 ES cells are transcriptionally more similar to wild-type ES cells than EpiSCs, differentiated EBs, or MEFs. Interestingly, we found that expression of a subset of STAT3 targets are downregulated in LIF-independent iOCT4 ES cells relative to wild-type ES cells, while a subset of OCT4/STAT3 co-occupied targets are upregulated. These results suggest that external activation of LIF-STAT3 signaling by addition of LIF in the culture media may drive a transcriptional network that cooperates with OCT4 to promote self-renewal. However, in the absence of LIF, OCT4 is capable of promoting self-renewal by regulating expression of a subnetwork of OCT4/STAT3 targets. The intersection of transcriptional networks between LIF-independent and wild-type ES cells may represent a minimal ground state network required for ES cell self-renewal. We also demonstrate that LIF-independent iOCT4 ES cells are epigenetically more similar to wild-type ES cells relative to EpiSCs or MEFs. Our epigenomic analyses revealed similar global profiles of histone modifications and active enhancers between LIF-independent and wild-type ES cells. In contrast, EpiSCs and MEFs displayed a different global profile of histone modifications. We also show that super-enhancers are similarly active between LIF-independent and wild-type ES cells, and we demonstrate that LIF-independent iOCT4 ES cells are fully capable of differentiating upon downregulation of OCT4. Upon addition of doxycycline and without LIF, LIF-independent iOCT4 ES cells form EBs and teratomas which contain cells represented in the three germ layers. However, while we found that LIF-independent iOCT4 ES cells are capable of homing to the ICM of mouse blastocysts, we did not evaluate the ability of LIF-independent iOCT4 ES cells to contribute to later stages of mouse chimera development, which is the most stringent assay for evaluating pluripotency of mouse ES cells.

**Figure 9:**
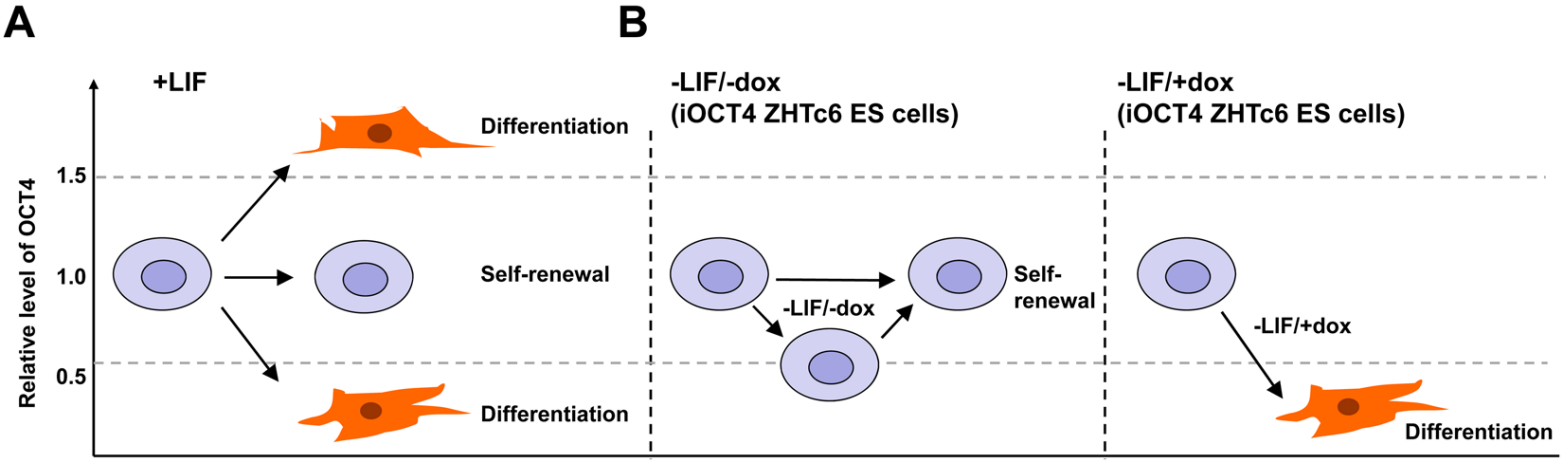
Model for relationship between OCT4 expression level and ES cell fate (**A**) In the presence of LIF, to maintain ES cell self-renewal, OCT4 expression must maintain a level of 50% to 150% normal diploid expression^5^. An increase in OCT4 expression above the threshold results in differentiation while a decrease below the threshold also leads to differentiation. (**B**) In the absence of LIF and doxycyline, self-renewal of ZHTc6 (iOCT4) ES cells is maintained when OCT4 expression is at a level of 50% to 100% normal diploid expression. In the absence of LIF and in the presence of doxycyline, ZHTc6 (iOCT4) ES cells undergo differentiation following downregulation of OCT4 expression.

There are several plausible mechanisms for how forced expression of OCT4 may facilitate ES cell self-renewal in the absence of LIF. First, the level of OCT4 expression may act as a molecular rheostat to regulate pluripotency versus differentiation. In support of this model, previous results showed that in the presence of LIF there is a dose-dependent effect of OCT4 on self-renewal and differentiation, where overexpression of OCT4 in ZHTc6 ES cells in the presence of LIF leads to differentiation^5^, while at half-dose OCT4 maintains ES cell self-renewal (Fig. 9A). However, in the absence of LIF, forced expression of OCT4 following doxycycline removal in ZHTc6 ES cells may instruct the cells to resist differentiation (Fig. 9B). Our results show that the level of OCT4 protein in LIF-independent iOCT4 ES cells cultured in the absence of LIF and doxycycline is ~69–80% that of control ES cells cultured in the presence of LIF and doxycycline (Supplemental Fig. 2A). These results demonstrate that OCT4 protein levels in LIF-independent iOCT4 (ZHTc6) ES cells are relatively similar in comparison to control ES cells (Fig. 3B, Supplemental Fig. 2A). While OCT4 transgene expression alone is not sufficient to maintain LIF-independent self-renewal (Figure 1C)^5^, exogenous OCT4 expression in combination with a wild-type endogenous OCT4 allele may facilitate ES cell self-renewal in the absence of LIF. ZHBTc4 ES cells, which express transgene-derived OCT4, but lack endogenous OCT4 expression, are unable to maintain long-term self-renewal in the absence of LIF (Figure 1C)^5^. However, because iOCT4 (ZHTc6) ES cells possess one wild-type endogenous OCT4 allele in addition to a Tet-off OCT4 transgene, and OCT4 expression is regulated by a positive feedback loop where the OCT4:SOX2 heterodimer binds to the OCT4 distal enhancer (DE)^52^, this autoregulatory circuit may promote self-renewal through the combined effect of exogenous and endogenous OCT4 expression. Second, because STAT3 is an upstream regulator of OCT4 expression, forced expression of exogenous OCT4 expression in combination with a wild-type endogenous OCT4 allele may essentially bypass the requirement for a LIF-centric self-renewal network. In support of this model, STAT3 binding nearby the OCT4 DE and promoter^9^ has been shown to regulate OCT4 expression^53^. In this case, OCT4 may compensate for LIF-independent activation of self-renewal transcriptional networks by binding to and regulating expression of OCT4/STAT3 co-occupied target genes. Our global expression profiling results support this model, where we observed downregulation of STAT3 target genes, and upregulation of OCT4/STAT3 target genes. Moreover, treatment with the JAK kinase inhibitor (JAKi), which blocks STAT3 phosphorylation, did not ablate self-renewal of LIF-independent iOCT4 ES cells, suggesting that exogenous and endogenous OCT4 expression may bypass the requirement for a LIF-centered self-renewal network. Third, OCT4 may drive a minimal transcriptional network that sustains ES cell self-renewal. In this case, as it has been demonstrated that STAT3 and OCT4 co-occupy a number of target genes^9^, partial functional redundancy may exist between an externally activated LIF-STAT3 signaling network and an OCT4-driven intrinsic network.

It has been shown that ERK phosphorylates human OCT4 at several residues^54–57^, and FGF/ERK signaling is critical for human ES cell self-renewal^58,59^. While several studies have evaluated the phosphorylation status of OCT4 in human ES cells, murine ES cell OCT4 phosphosites are largely unknown. While FGF/ERK signaling is critical for human ES cell self-renewal, inhibition of MEK and FGFR^10^ promotes ES cell self-renewal. LIF withdrawal has been shown to activate ERK^60^, and ERK signaling suppresses ES cell self-renewal. However, it is not clear whether ERK phosphorylates mouse OCT4 upon LIF withdrawal. It has been suggested that a site in the homeobox domain of murine OCT4 in P19 cells is phosphorylated based on a predictive algorithm^61^. However, this posttranslational modification has not been directly identified in mouse pluripotent ES cells. To investigate the phosphorylation status of OCT4 in LIF-independent iOCT4 ES cells we performed mass spectrometry (LC/MS/MS) using nuclear extracts from wild-type and LIF-independent iOCT4 ES cells. While we observed N-terminal acetylation of OCT4 in wild-type (R1) ES cells cultured in self-renewal conditions (+LIF/+GSK3i), and N-terminal acetylation of OCT4 in two iOCT4 ES cell lines cultured in LIF-independent media (−LIF/+GSK3i/−dox), we did not identify OCT4 phosphosites in wild-type or iOCT4 ES cells, suggesting that OCT4 is not inactivated in LIF-independent iOCT4 ES cells.

While OCT4 is a key transcriptional regulator of the ES cell self-renewal network, OCT4 mRNA and protein levels have been shown to persist following 3–4 days of differentiation^62,63^. There are several plausible scenarios for the perplexing delay in downregulation of OCT4 during differentiation. First, because multiple transcription factors (e.g. ESRRB, OCT4, SOX2, KLF4, etc) have been shown to occupy promoter and enhancer regions of OCT4^9^, it is possible that several of these OCT4-binding transcription factors, although downregulated during differentiation, may be sufficient at reduced expression levels to act in combination to drive expression of OCT4 for several days. In this case, it is possible that differentiation may commence following downregulated expression of a network of pluripotency-regulators that act on OCT4 regulatory sequences, which subsequently leads to downregulated expression of OCT4 and a full collapse of the ES cell transcriptional network. Second, OCT4 may partner with differentiation-inducing transcriptional regulators to promote differentiation. Altogether, our findings provide novel insight into the role for OCT4 in sustaining ES cells self-renewal in the absence of LIF.

## Materials and Methods

### ES cell culture

ES cells were cultured as previously described with minor modifications^25,37^. Briefly, wild-type (R1), ZHTc6 and ZHBTc4 ES cells^5^ were cultured on gelatin-coated dishes in ESC media containing DMEM/15% FBS media containing LIF (ESGRO) and 2 µg/mL doxycycline at 37 °C with 5% CO_2_, and 1.5 µM CHIR9901 (GSK3 inhibitor) or 1.5 µM CHIR9901/1 µM MEK inhibitor (2i). For derivation of LIF-independent iOCT4 ES cells, iOCT4 (ZHTc6) ES cells were cultured in FBS-containing ESC media without LIF or doxycycline, and with 1.5 µM CHIR9901 (GSK3i) for up to two weeks. Individual ESC clones were subsequently picked, washed in PBS, dissociated with trypsin, and expanded for further characterization. For LIF-independent culture, iOCT4 (ZHTc6) ES cells were cultured in 1) FBS-containing ESC media without doxycycline or LIF, and with 1.5 µM CHIR9901 or 2i, 2) FBS-containing ESC media without doxycycline, LIF, or CHIR9901 (GSK3i), or 3) serum-free defined ES cell media (N2B27)^10^ without LIF or CHIR9901. For LIF-independent culture of ES cells at clonal density, 500 ES cells were cultured in serum-free or FBS-containing media in a 6-well with or without JAK inhibitor I (Cayman chemical; 0.6 µM^18^). For ES cells cultured in FBS-containing media, ES cells were passed by washing with PBS, and dissociating with trypsin. For ES cells cultured in serum-free media, ES cells were passed by washing with PBS, and dissociating with accutase.

For embryoid body (EB) formation, ES cells were cultured in low attachment binding dishes to promote 3D formation in ES cell media without LIF but with doxycycline. Alkaline phosphatase staining was performed using a kit from Millipore according to the manufacturer’s instructions. For immunofluorescence analysis following differentiation, ES cells were cultured on gelatin-coated chamber slides in serum-free or FBS-containing ES cell media without LIF but with doxycycline and with or without retinoic acid.

### Chromosome counting

Chromosome counting was performed as previously described^64^. Briefly, ES cells were cultured on 0.1% gelatin-coated dishes in ES culture media. Colcemid (0.06 µg/mL) was added to the media and ES cells were incubated at 37 °C for 3 h. ES cells were pelleted and subsequently incubated with KCl at room temperature and then incubated with a cold fixative solution (3 methanol:1 glacial acetic acid). Fixed cells were dropped on slides and allowed to dry overnight at 40 °C. The next day slides were stained with Giemsa dye (KaryoMax/GURR tablets), and chromosomes were counted using standard microscopy.

### EpiSC culture

E3 EpiSCs were culture as previously described with minor modifications^65^. Briefly, EpiSCs were cultured in EpiSC media containing Knockout DMEM supplemented with 20% Knockout serum replacement, L-glutamine, pen/strep, and bFGF^65,66^. EpiSCs were passed by washing with PBS, and dissociating with 0.05% EDTA. Following passage, EpiSCs were transiently cultured with 10 µM ROCK inhibitor (Y27632).

### Immunofluroescence analysis

ES cells were fixed with 4% paraformaldehyde for 15 min at room temperature, washed three times with 0.1% Triton X-100 (Sigma), and blocked in 1% BSA/0.01% Tween-20/PBS for 30 min. The fixed cells were then incubated with a primary-conjugated antibody overnight at 4 °C in blocking buffer. The next day, the cells were washed three times with blocking buffer for 15 min.

### Cell cycle analysis

Cell cycle analysis was performed as previously described^67^. Briefly, cells were trypsinized, washed in PBS, fixed in 70% ethanol, and stained with DAPI/Triton X-100 staining solution (0.1% Triton X-100/1 µg/mL DAPI). FACS analysis was subsequently performed at the Wayne State University Microscopy, Imaging, and Cytometry core facility.

### Teratoma formation

ES cells were cultured on gelatin coated dishes to remove feeder cells, dissociated into single cells, and 10^6^ ES cells were injected subcutaneously into SCID-beige mice, which we administered tetracycline in their water. After three to four weeks, mice were euthanized and teratomas were washed and fixed in 10% buffered formalin. Teratomas were then embedded in paraffin. Thin sections were cut and stained with hematoxylin and eosin (H&E) using standard techniques. All animals were treated in accordance with Institution Animal Care and Use Committee guidelines under approved protocols at NHLBI.

### Nuclear extraction and western blotting

Nuclear extracts were prepared from ES cells using a standard high salt extraction protocol^68^. Briefly, cells were lysed by Dounce homogenizing in buffer A, washed, and nuclear proteins were extracted with buffer C. OCT4 nuclear protein levels were analyzed by SDS PAGE. The phospho-STAT3 (pY705) antibody was obtained from Cell Signaling (#9131).

### ChIP-Seq analysis

ChIP-Seq experiments were performed as previously described with minor modifications^25,37,38,69^. The monoclonal H3K4me3 antibody (CS200580) was obtained from Millipore, the monoclonal H3K4me2 (ab32356), the polyclonal H3K27ac (ab4729), and monoclonal H3K27me3 (ab6002) antibodies were obtained from Abcam. Briefly, 10^7^–10^8^ mouse ES cells were harvested and chemically crosslinked with 1% formaldehyde (Sigma) for 5–10 minutes at 37 °C and subsequently sonicated. Sonicated cell extracts equivalent to 5 × 10^6^ cells were used for ChIP assays. ChIP-enriched DNA was end-repaired using the End-It DNA End-Repair kit (Epicentre), followed by addition of a single A nucleotide, and ligation of custom Illumina adapters. PCR was performed using Phusion High Fidelity PCR master mix. ChIP libraries were sequenced on Illumina HiSeq platforms according to the manufacture’s protocol. Sequence reads were mapped to the mouse genome (mm9) using bowtie2^70^ with default settings. ChIP-Seq read enriched regions were identified by SICER^47^ with a window size setting of 200 bps, a gap setting of 400 bps and a FDR setting of 0.001. The RPBM measure (read per base per million reads) was used to quantify the density of histone modifications at genomic regions from ChIP-Seq datasets.

### RNA-Seq analysis

RNA-Seq was performed as previously described^25,37^. Poly-A mRNA was purified using a Dynabeads mRNA purification kit. Double-stranded cDNA was generated using a Super-Script double-stranded cDNA synthesis kit (Invitrogen). cDNA was subjected to library preparation as described above. RNA-Seq libraries were sequenced on an Illumina HiSeq platform according to the manufacturer’s protocol.

The RPKM measure (read per kilobases of exon model per million reads)^71^ was used to quantify the mRNA expression level of a gene from RNA-Seq data. Differentially expressed genes were identified using edgeR (false discovery rate [FDR] <0.001; fold-change [FC] >1.5)^72^.

### Generation of ES chimeras

#### Lentiviral Transduction

LIF-independent iOCT4 ES cells were transduced with lentiviral particles encoding GFP as previously described with minor modifications^37^. To generate lentiviral particles, HEK 293 T cells were co-transfected with an envelope plasmid (pLP/VSVG), packaging vector (psPAX2), and a GFP expression vector using lipofectamine 2000. Twenty-four to 48 hrs post transfection, the medium containing lentiviral particles was harvested and used to infect LIF-independent iOCT4 ES cells. Twenty-four hours post transduction ES cells were stably selected in the presence of 1 µg/mL puromycin. Blastocyst injection: E3.5 blastocysts were flushed from the uteri of CD1 female mice using M2 media, in accordance with Institution Animal Care and Use Committee guidelines under current approved protocols at Wayne State University. ES blastocyst chimeras were generated by microinjecting 10–15 LIF-independent iOCT4 ES cells into E3.5 CD1 blastocysts. Injected blastocysts were cultured in KSOM media and incubated overnight at 37 °C with 5% CO_2_.

## Electronic supplementary material

Supplemental Movie 1

Supplemental Movie 2

Supplementary Information
