# Supplementary material for: OCT4 supports extended LIF-independent self-renewal and maintenance of transcriptional and epigenetic networks in embryonic stem cells
Source: Sci Rep. 2017 Nov 27;7:16360. doi: 10.1038/s41598-017-16611-y (PMC5703885; doi:10.1038/s41598-017-16611-y)
Supplement: Supplementary file 3 — Supplementary Information [file 41598_2017_16611_MOESM3_ESM.pdf]

SUPPLEMENTAL INFORMATION

OCT4 supports extended LIF-independent self-renewal and maintenance of transcriptional and epigenetic networks in embryonic stem cells

Runsheng He[1-2], Besa Xhabija[1-2], Batool Al-Qanber[1-2], Benjamin L. Kidder[1-2*]

[1]Department of Oncology, Wayne State University School of Medicine, Detroit, MI, USA

[2]Karmanos Cancer Institute, Wayne State University School of Medicine, Detroit, MI, USA

Supplemental Figure 1. OCT4 overexpression promotes LIF-independent ES cell self-renewal in the absence of GSK3i and with or without FBS

AP staining of (**A**) wild-type ES cells and (**B-C**) LIF-independent iOCT4 ES cells cultured in LIF-independent ES cell media containing FBS, without GSK3i, and with JAKi (bottom) or without JAKi (top) for 12 days. **(D)** ES cells were scored by AP staining. The percentage of AP positive colonies is represented as mean ± SEM. p values were calculated using a t test. **(E)** Bright-field microscopy (top) and AP staining (bottom) of wild-type ES cells cultured in LIF-independent serum-free ES cell media without GSK3i for 7 days. **(F-G)** Bright-field microscopy (top) and AP staining (bottom) of iOCT4 ES cells cultured in LIF-independent serum-free ES cell media without GSK3i or doxycycline for 7 days. **(H)** ES cells were scored by AP staining. The percentage of AP positive colonies is represented as mean ± SEM. p values were calculated using a t test. (**I-K**) AP staining of (**I**) wild-type and (**J-K**) LIF-independent iOCT4 ES cells cultured at a moderate cell density (2,500 cells / 6-well) in serum-free and LIF-independent ES cell media with JAKi (bottom) or without JAKi (top).

Supplemental Figure 2. Expression of OCT4, SOX2, and STAT3 in LIF-independent iOCT4 ES cells

(**A-D**) Western blot images with original exposure. (**A**) Western blots of OCT4 and SOX2, and STAT3 and STAT3-pY705, in control (ZHTc6) ES cells and LIF-independent iOCT4 ES cells cultured in FBS-containing media and GSK3i. HSC70 or ACTIN were used as loading controls. OCT4 levels were normalized to HSC70 using

ImageJ software (https://imagej.nih.gov/ij/). **(B)** Western blot of OCT4 and SOX2, and STAT3 and STAT3-pY705, in wild-type (R1) and iOCT4 ES cells cultured in LIF-independent serum-free ES cell media without GSK3i or doxycycline. OCT4 levels were normalized to HSC70 using ImageJ software (https://imagej.nih.gov/ij/). **(C)** Western blot of OCT4, and STAT3 and STAT3-pY705, in LIF-independent iOCT4 ES cells cultured without GSK3i or LIF and with doxycycline to repress OCT4 transgene expression over a time-course of 6 days. Note that the levels of OCT4 and STAT3-pY705 protein is significantly decreased after three days of culture in the presence of doxycycline. (**D**) Bright-field microscopy of wild-type ES cells cultured in LIF-independent ES cell media with doxycycline but without GSK3i for 3 and 6 days. **(E)** Western blot of OCT4 in wild-type (R1) ES cells cultured in FBS-containing ES cell media with or without LIF, and with or without GSK3i for 11 days. Note that the level of OCT4 protein is significantly reduced in wild-type ES cells cultured in the absence of LIF.

Supplemental Movie 1. Video 1 of spontaneously beating cardiomyocyte-like cells. Wild-type R1 ES cells were cultured in serum-free media in the absence of LIF for two weeks to induce differentiation.

Supplemental Movie 2. Video 2 of spontaneously beating cardiomyocyte-like cells. LIF-independent iOCT4 ES cells were cultured in serum-free media without LIF in the presence of doxycycline for two weeks to induce differentiation.

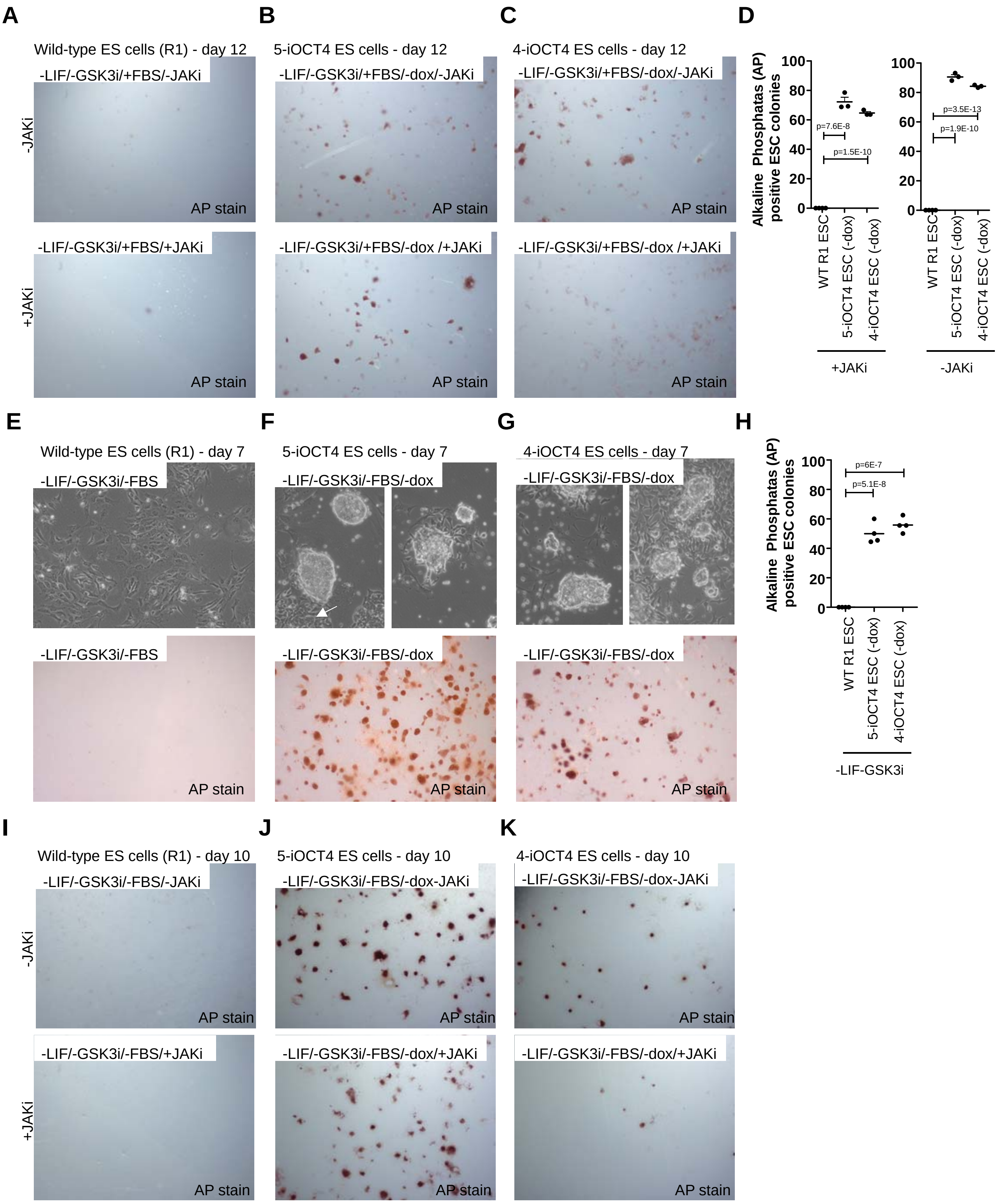

Supplemental Figure 1

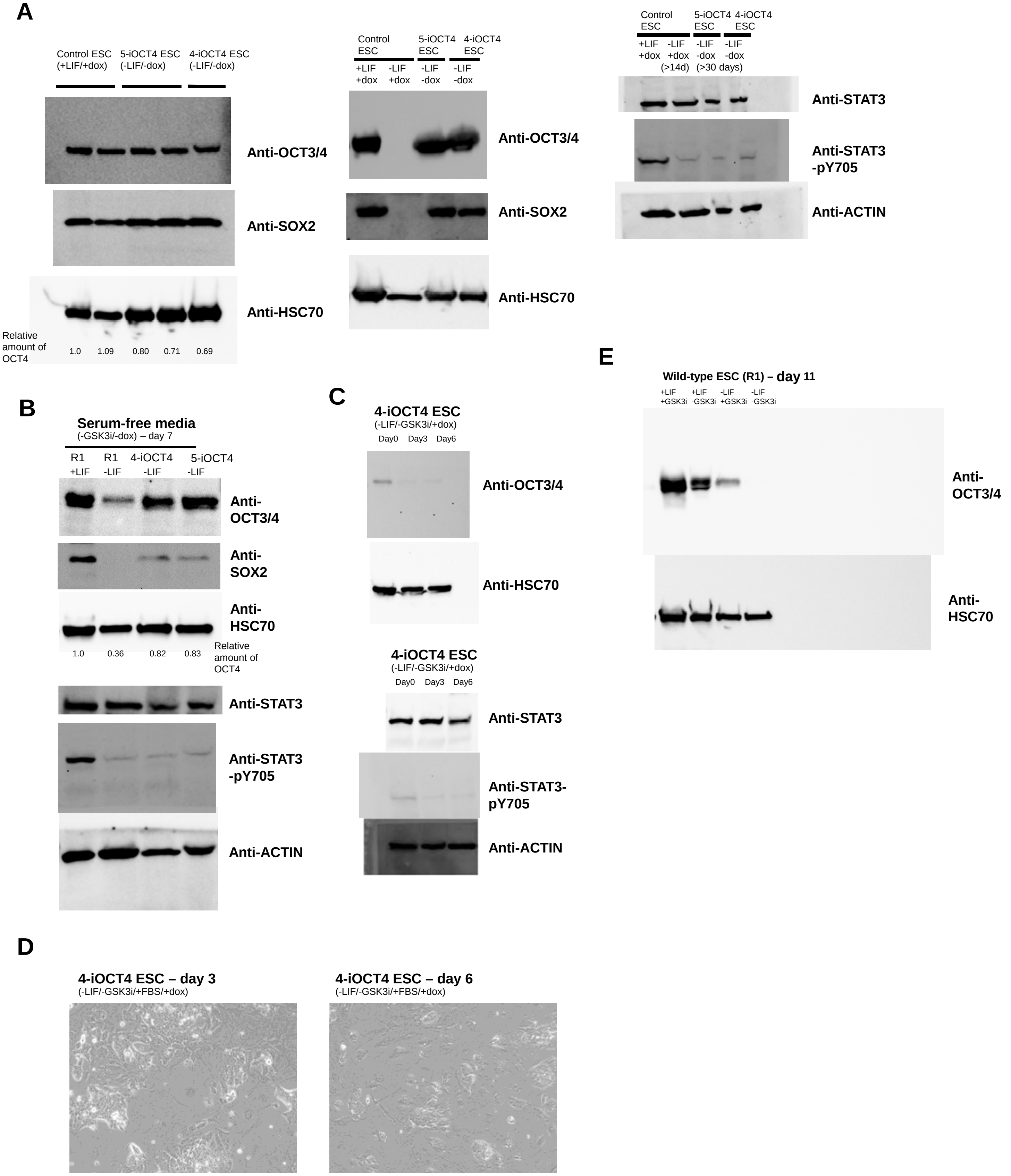

Supplemental Figure 2
